# MIMO Gaussian State-Dependent Channels with a State-Cognitive Helper

**DOI:** 10.3390/e21030273

**Published:** 2019-03-12

**Authors:** Michael Dikshtein, Ruchen Duan, Yingbin Liang, Shlomo Shamai (Shitz)

**Affiliations:** 1 Department of Electrical Engineering, Technion-Israel Institute of Technology, Haifa 32000, Israel; 2 Samsung Semiconductor Inc., San Jose, CA 95134, USA; 3 Department of ECE, The Ohio State University, Columbus, OH 43210, USA

**Keywords:** dirty paper coding, Gel’fand–Pinsker scheme, non-causal channel state information, network information theory

## Abstract

We consider the problem of channel coding over multiterminal state-dependent channels in which neither transmitters nor receivers but only a helper node has a non-causal knowledge of the state. Such channel models arise in many emerging communication schemes. We start by investigating the parallel state-dependent channel with the same but differently scaled state corrupting the receivers. A cognitive helper knows the state in a non-causal manner and wishes to mitigate the interference that impacts the transmission between two transmit–receive pairs. Outer and inner bounds are derived. In our analysis, the channel parameters are partitioned into various cases, and segments on the capacity region boundary are characterized for each case. Furthermore, we show that for a particular set of channel parameters, the capacity region is entirely characterized. In the second part of this work, we address a similar scenario, but now each channel is corrupted by an independent state. We derive an inner bound using a coding scheme that integrates single-bin Gel’fand–Pinsker coding and Marton’s coding for the broadcast channel. We also derive an outer bound and further partition the channel parameters into several cases for which parts of the capacity region boundary are characterized.

## 1. Introduction

**Figure 1:**
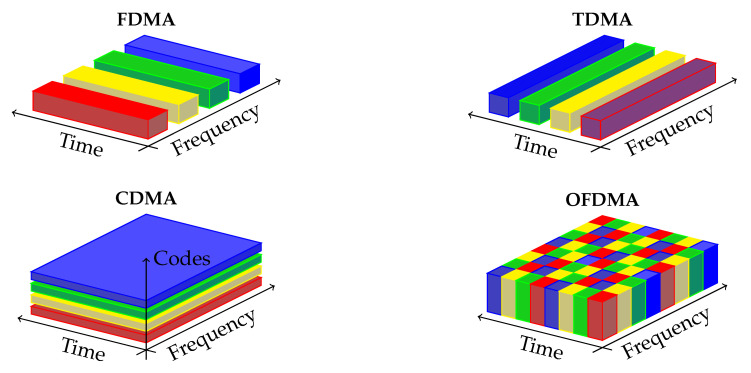
Orthogonal Multiple Access Techniques.

Cellular communication systems are designed to allow multiple users to share the same communication medium. Traditionally, mobile networks have enabled this feature by dividing the physical resources (such as time, frequency, code, and space) in an orthogonal manner between users. An illustration of the typical methods, called Orthogonal Multiuser Access (OMA) is shown in Figure 1.

The future of cellular communications is facing exponential growth in bandwidth demand. Furthermore, increased popularity in Internet of Things (IoT) applications and the emergence of Vehicle-to-Vehicle (V2V) connectivity will further grow the number of network consumers. Hence, fifth-generation (5G) wireless networks are required to support extensive connectivity, low latency, and higher data rates. Such requirements cannot be satisfied using the traditional OMA methods and thus to sustain more users and higher transmission rates, non-orthogonal multiuser access (NOMA) has been intensively investigated, where interference mitigation is the key issue for non-orthogonal transmission. A comprehensive survey on NOMA from an information theoretic perspective is given in [1].

**Figure 2:**
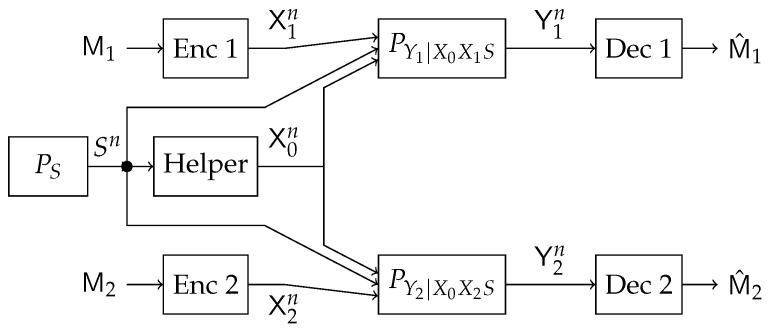
General State-Dependent Parallel Channel with a helper.

**Figure 3:**
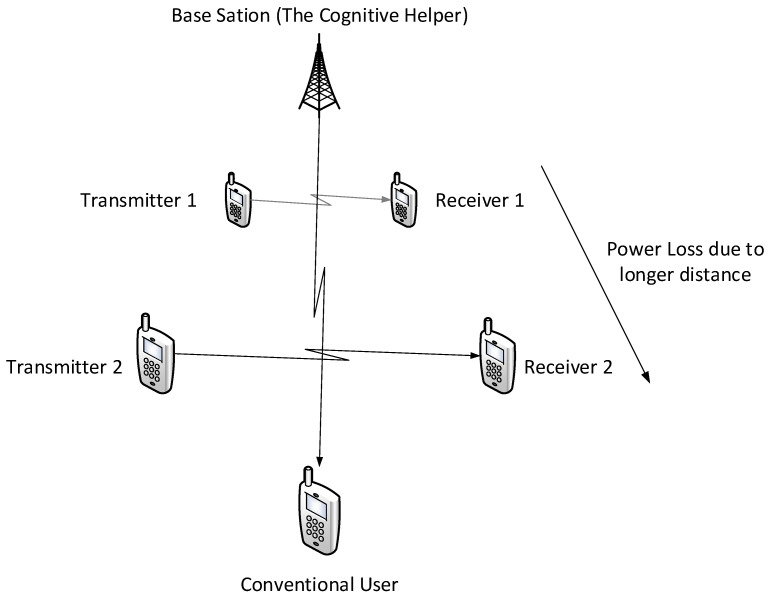
Particular NOMA configuration.

In this work, we study a particular communication model that can be used in future NOMA techniques. Specifically, we investigate a type of state-dependent channel with a helper, illustrated in Figure 2, in which two transmitters wish to send messages to their corresponding receivers over a parallel state-dependent channel. The state is not known to either transmitter or receiver but is non-causally (the side information in all times is given to the encoder before the block transmission) known to a state-cognitive helper, who tries to assist each receiver in mitigating the interference caused by the state. This model captures interference cancelation in various practical scenarios. For example, users in multi-cell systems may be interfered by a base station located in other cells. Such a base station, being as the source that causes the interference, clearly knows the information of the interference (modeled by state) and can serve as a helper to mitigate the interference. Alternatively, that base station can also convey the interference information to other base stations via the backhaul network so that other base stations can serve as helpers to reduce the interference. As another example, consider a situation where there are two Device to Device (D2D) links located in two distinct cells, and there is a downlink signal sent from the base station to some conventional mobile user in the cell. Also, there is some central unit that knows in a non-causal manner the signal to be sent by each base station, the helper in our model, and tries to assist the D2D communication links by mitigating the interference (see Figure 3). As a comparison, this type of state-dependent models differs from the original state-dependent channels studied in, e.g., [2,3], in that the state-cognitive helper is not informed of the transmitters’ messages, and hence its state cancelation strategies are necessarily independent of message encoding at the transmitters.

The study of channel coding in the presence of channel side information (CSI) was initiated by Shannon [4] who considered a discrete memoryless channel (DMC) channel with random parameters and side information provided causally to the transmitter. The single-letter expression for the capacity of the point-to-point DMC with non-causal CSI at the encoder (the G-P channel) was derived in the seminal work of Gel’fand and Pinsker [2]. One of the most interesting special cases of the G-P channel is the Gaussian additive noise and interference setting in which the additive interference plays the role of the state sequence, which is known non-causally to the transmitter. Costa showed in [3] that the capacity of this channel is equal to the capacity of the same channel without additive interference. The capacity achieving scheme of [3] (which is that of [2] applied to the Gaussian case) is termed “writing on dirty paper” (WDP), and consequently, the property of the channel where the known interference can be completely removed is dubbed “the WDP property”. Cohen and Lapidoth [5] showed that any interference sequence can be removed entirely when the channel noise is ergodic and Gaussian.

The models we study in this work all have a broadcasting node. The *discrete memoryless broadcast channel* (DM-BC) was introduced by Cover [6]. The capacity region of the DM-BC is still an open problem. The largest known inner bound on the capacity region of the DM-BC with private messages was derived by Marton [7]. Liang [8] derived an inner bound on the capacity region of the DM-BC with an additional common message. The best outer bound for DM-BC with a common message is due to Nair and El Gamal [9]. There are, however, some special cases where the capacity region is fully characterized. For example, the capacity region of the degraded DM-BC was established by Gallager [10]. The capacity region of the Gaussian BC was derived by Bergmans [11]. An interesting result is the capacity region of the Gaussian MIMO BC which was established by Weingarten et al. [12]. The authors introduced a new notion of *an enhanced channel* and used it jointly with the Entropy Power Inequality (EPI) to show their result. The capacity achieving scheme relies on the dirty paper coding technique. Liu and Viswanath [13] developed *an extremal inequality* proof technique and showed that it can be used to establish a converse result in various Gaussian MIMO multiterminal networks, including the Gaussian MIMO BC with private messages. Recently, Geng and Nair [14] developed a different technique to characterize the capacity region of Gaussian MIMO BC with common and private messages.

Degraded DM-BC with causal and non-causal side information was introduced by Steinberg [15]. Inner and outer bounds on the capacity region were derived. For the particular case in which the nondegraded user is informed about the channel parameters, it was shown that the bounds are tight, thus obtaining the capacity region for that case. The general DM-BC with non-causal CSI at the encoder was studied by Steinberg and Shamai [16]. An inner bound was derived, and it was shown to be tight for the Gaussian BC with private messages and independent additive interference at both channels. The latter setting was recently extended to the case of common and private messages in the Gaussian framework with *K* users in [17]. The special case where the transmitter sends only a common message to all receivers over an additive BC has been initially studied in [18] and has been recently extended to the compound setting in [19]. Outer bounds for DM-BC with CSI at the encoder were derived in [20].

The models addressed in this paper have a mismatched property, that is the state sequence is known only to some nodes, which differs from the classical study on state-dependent channels. The type of channels with mismatched property has been addressed in the past for various models, for example, in [21,22,23,24,25], the state-dependent *multiple access channel* (MAC) is studied with the state known at only one transmitter. The best outer bound for the Gaussian MAC setting was recently reported in [26]. The point-to-point helper channel studied in [27,28] can be considered as a special case of [25], where the cognitive transmitter does not send any message. Further in [28], the state-dependent MAC with an additional helper was studied, and the partial/full capacity region was characterized under various channel parameters. Moreover, some state-dependent relay channel models can also be viewed as an extension of the state-dependent channel with a helper, where the relay serves the role of the helper by knowing the state information. In [29], the state-dependent relay channel with state non-causally available at the relay is considered. An achievable rate was derived using a combination of decode-and-forward, Gel`fand–Pinsker (GP) binning and codeword splitting. Also, in [30], additional noiseless cooperation links with finite capacity were assumed between the transmitter and the relay, and various coding techniques were explored. The authors of [31] have recently considered a different scenario with a state-cognitive relay. The state-dependent Z-IC with a common state known in a non-causal manner only to the primary user was studied in [32]. A good tutorial on channel coding in the presence of CSI can be found in [33].

**Figure 4:**
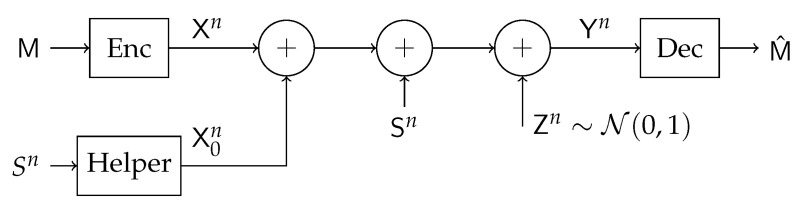
Point-to-Point Helper Channel.

The basic state-dependent Gaussian channel with a helper is illustrated in Figure 4. It was first introduced in [27], where the capacity in the infinite power regime was characterized and was shown to be achievable by lattice coding. The capacity under arbitrary state power was established for some special cases in [28]. Based on a single-bin GP binning scheme the following lower bound was derived for the discrete memoryless case

$$R\leq \max_{P_{UX_{0}|S}P_{X}}\min\left\{I\left(X;Y|U\right),I\left(\mathrm{UX};Y\right)-I\left(U;S\right)\right\}.$$

This lower bound was further evaluated for Gaussian channel by appropriate choice of the maximizing input distribution. The surprising result of that study was that when the helper power is above some threshold, then the interference caused by the state is entirely canceled and the capacity of the channel without the state can be achieved. This threshold does not depend on the state power, and hence it was shown that this channel also has WDP property, that is the capacity of the channel is the same as the capacity of the similar channel without the interference (which is modeled as the state).

The most relevant work to this study is [34], in which the state-dependent parallel channel with a helper was studied, for the regime with infinite state power and with two receivers being corrupted by two independent states. A time-sharing scheme was proved to be capacity achieving under certain channel parameters. In contrast, in this study, we expand those results for the arbitrary state power regime. We also consider two extreme cases. At first, we address the problem where the two receivers of the parallel channel are corrupted by the same but differently scaled states, and in the second part, those states are independent. For both cases, we show that the time-sharing scheme is no longer optimal. Our main contribution in this work is a derivation of inner bound, which is an extension of the Marton coding scheme for the discrete broadcast channel to the current model. We will apply this bound for the MIMO Gaussian setting and characterize the segments of the capacity region for various channel parameters. The material in this paper was presented in part at [35,36].

## 2. Preliminaries

### 2.1. Notation Conventions

Throughout the paper, random variables are denoted using a sans-serif font, e.g., X, their realizations are denoted by the respective lower-case letters, e.g., *x*, and their alphabets are denoted by the respective calligraphic letters, e.g., X. Let $X^{n}$ stand for the set of all *n*-tuples of elements from X. An element from $X^{n}$ is denoted by $x^{n}=\left(x_{1},x_{2},\cdots ,x_{n}\right)$ and substrings are denoted by $x_{i}^{j}=\left(x_{i},x_{i+1},\cdots ,x_{j}\right)$. The cardinality of a finite set, say X, is denoted by |X|. The probability distribution function of X, the joint distribution function of X and Y, and the conditional distribution of X given Y are denoted by $P_{X}$, $P_{X,Y}$ and $P_{X|Y}$ respectively. The expectation of X is denoted by $E_{}\left[X\right]$. The probability of an event E is denoted as P{E}. The set of jointly ϵ-typical *n*-tuples $\left(x^{n},y^{n}\right)$ is defined as $T_{\epsilon }^{\left(n\right)}\left(P_{XY}\right)$ [37]. A set of consecutive integers starting at 1 and ending in $\left\lceil 2^{nR}\right\rceil$ is denoted as $I_{R}^{\left(\;n\;\right)}≜\left\{1,2,\cdots ,\left\lceil 2^{nR}\right\rceil \right\}$. We assume throughout this paper that $2^{nR}$ are integers, for any *R* and n→∞.

We denote the covariance of a zero mean vector X by $\Sigma_{X}≜E_{}\left[XX^{T}\right]$, $\Sigma_{XY}≜E_{}\left[XY^{T}\right]$ is the cross-correlation, and the conditional correlation matrix of X given Y as $M_{X|Y}≜\Sigma_{X}-\Sigma_{XY}\Sigma_{Y}^{-1}\Sigma_{YX}$.

### 2.2. Definitions

**Definition** **1.** *Random variables X, Y, Z are said to form a Markov chain in that order (denoted by X→Y→Z) if the conditional distribution of Z depends only on Y and is conditionally independent of X. Specifically, X, Y and Z form a Markov chain X→Y→Z if the joint probability mass function can be written as*

(1) $$P_{XYZ}=P_{X}P_{Y|X}P_{Z|Y}.$$

### 2.3. Auxiliary Results

This section introduces some auxiliary results that are relevant to the analysis in this work [37].

**Lemma** **1** (Data-processing inequality). *If X→Y→Z, then*

(2) I(X;Y)≥I(X;Z).

The following inequality will be frequently used in the proofs of outer bounds on the capacity regions.

**Lemma** **2** (Fano’s Inequality). *Let $\left(X,Y\right)∼P_{XY}$ and $P_{e}=\mathrm{Pr}\left(X\neq Y\right)$. Then*

(3) $$H\left(X|Y\right)\leq H\left(P_{e}\right)+P_{e}\log|X|\leq 1+P_{e}\log\left|X\right|.$$

The covering lemma and the packing lemma will be used in the achievability proofs throughout this paper.

**Lemma** **3** (Covering Lemma). *Let $\left(U,X,\hat{X}\right)∼P_{UX\hat{X}}$ and $\epsilon^{\prime }<\epsilon$. Let $\left(U^{n},X^{n}\right)∼P_{U^{n}X^{n}}$ be a pair of random sequences with*

$$\lim_{n\to \infty }P\left\{\left(U^{n},X^{n}\right)\in T_{\epsilon^{\prime }}^{\left(n\right)}\left(P_{UX}\right)\right\}=1,$$

*and let ${\hat{X}}^{n}\left(m\right)$, m∈A, where $\left|A\right|\geq 2^{nR}$, be random sequences, conditionally independent of each other and of $X^{n}$ given $U^{n}$, each distributed according to $\prod_{i=1}^{n}P_{\hat{X}|U}\left({\hat{x}}_{i}|u_{i}\right)$. Then, there exists δ(ϵ) that approaches zero as ϵ→0 such that*

(4) $$\lim_{n\to \infty }P\left\{\left(U^{n},X^{n},{\hat{X}}^{n}\left(m\right)\right)\notin T_{\epsilon }^{\left(n\right)}\;\mathrm{for}\;\mathrm{all}\;m\in A\right\}=0,$$

*if $R>I\left(X;\hat{X}|U\right)+\delta \left(\epsilon \right)$.*

**Lemma** **4** (Packing Lemma). *Let $\left(U,X,Y\right)∼P_{UXY}$. Let $\left({\tilde{U}}^{n},{\tilde{Y}}^{n}\right)∼P_{{\tilde{U}}^{n}{\tilde{Y}}^{n}}$ be a pair of arbitrarily distributed random sequences, not necessarily distributed according to $\prod_{i=1}^{n}P_{UY}\left({\tilde{u}}_{i},{\tilde{y}}_{i}\right)$. Let $X^{n}\left(m\right)$, m∈A, where $\left|A\right|\leq 2^{nR}$, be random sequences, each distributed according to $\prod_{i=1}^{n}P_{X|U}\left(x_{i}|{\tilde{u}}_{i}\right)$. Further assume that $X^{n}\left(m\right)$, m∈A, is pairwise conditionally independent of ${\tilde{Y}}^{n}$ given ${\tilde{U}}^{n}$, but is arbitrarily dependent on other $X^{n}\left(m\right)$ sequences. Then, there exists δ(ϵ) that approaches zero as ϵ→0 such that*

$$\lim_{n\to \infty }P\left\{\left({\tilde{U}}^{n},X^{n}\left(m\right),{\tilde{Y}}^{n}\right)\in T_{\epsilon }^{\left(n\right)}\;\mathrm{for}\;\mathrm{some}\;m\in A\right\}=0,$$

*if R<I(X;Y|U)−δ(ϵ).*

## 3. The MIMO Gaussian Channel with Same but Differently Scaled States

### 3.1. Channel Model

**Figure 5:**
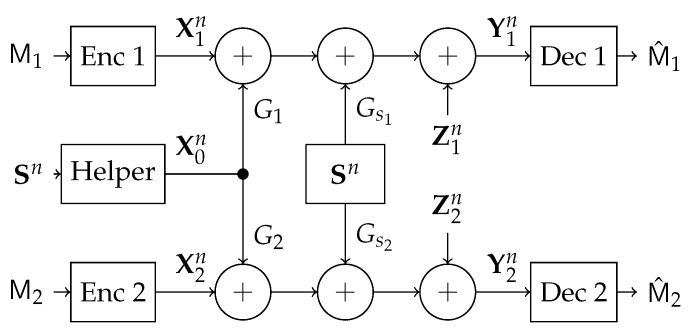
The state-dependent parallel channel with same but differently scaled states and a state-cognitive helper.

In this section, we study the state-dependent parallel network with a state-cognitive helper, in which two transmitters communicate with two corresponding receivers over a state-dependent parallel channel. The two receivers are corrupted by the same but differently scaled state, respectively. The state information is not known to either the transmitters or the receivers, but a helper non-causally. Hence, the helper assists these receivers to cancel the state interference (see Figure 5).

More specifically, the encoder at transmitter *l*, $f_{l}\;:\;I_{R_{l}}^{\left(\;n\;\right)}\to X_{l}^{n}$, maps a message $m_{l}\in I_{R_{l}}^{\left(\;n\;\right)}$ to a codeword $x_{l}^{n}$, for l=1,2. The inputs $x_{1}^{n}$ and $x_{2}^{n}$ are sent respectively over the two subchannels of the parallel channel. The two receivers are corrupted by the same but differently scaled and identically distributed (i.i.d.) state sequence $s^{n}\in S^{n}$, which is known to a common helper non-causally. Hence, the encoder at the helper, $f_{0}\;:\;S^{n}\to X_{0}^{n}$, maps the state sequence $s^{n}\in S^{n}$ into a codeword $x_{0}^{n}\in X_{0}^{n}$. The channel transition probability is given by $P_{Y_{1}|X_{0}X_{1}S}\cdot P_{Y_{2}|X_{0}X_{2}S}$. The decoder at receiver *l*, $g_{l}:Y_{l}^{n}\to I_{R_{l}}^{\left(\;n\;\right)}$, maps a received sequence $y_{l}^{n}$ into a message ${\hat{m}}_{l}\in I_{R_{l}}^{\left(\;n\;\right)}$, for l=1,2. We assume that the messages are uniformly distributed over the sets $I_{R_{1}}^{\left(\;n\;\right)}$ and $I_{R_{2}}^{\left(\;n\;\right)}$. We define the average probability of error for a length-*n* code as

(5) $$P_{e}^{\left(n\right)}=\frac{1}{2^{n(R_{1}+R_{2})}}\sum_{m_{1}=1}^{2^{nR_{1}}}\sum_{m_{2}=1}^{2^{nR_{2}}}P\left\{\left({\hat{m}}_{1},{\hat{m}}_{2}\right)\neq \left(m_{1},m_{2}\right)\right\}.$$

**Definition** **2.** *A rate pair $\left(R_{1},R_{2}\right)$ is said to be achievable if there exist a sequence of message sets $I_{R_{1}}^{\left(\;n\;\right)}$ and $I_{R_{2}}^{\left(\;n\;\right)}$, and encoder-decoder tuples $\left(f_{0}^{\left(n\right)},f_{1}^{\left(n\right)},f_{2}^{\left(n\right)},g_{1}^{\left(n\right)},g_{2}^{\left(n\right)}\right)$ such that the average probability of error $P_{e}^{\left(n\right)}\to 0$ as n→∞.*

**Definition** **3.** *We define the capacity region of the channel as the closure of the set of all achievable rate pairs $\left(R_{1},R_{2}\right)$.*

In this section, we focus on the MIMO Gaussian channel, with the outputs at the two receivers for one channel use given by

(6) $$Y_{l}=G_{l}X_{0}+X_{l}+G_{s_{l}}S+Z_{l}\;l\in \left\{1,2\right\},$$

where $X_{0}$, $X_{1}$, $X_{2}$, $S_{2}$, $Z_{1}$ and $Z_{2}$ are all real vectors of size t×1, and

- $X_{0}$, $X_{1}$, $X_{2}$ are the input vectors that are subject to the covariance matrix constraints $\frac{1}{n}\sum_{i=1}^{n}x_{li}x_{li}^{T}⪯K_{l}$, l∈{0,1,2},
- $Y_{l}$ is the output vector, l∈{1,2},
- S is a real Gaussian random vector with zero mean and covariance matrix $K_{S}=E_{}\left[SS^{T}\right]⪰0$,
- $Z_{l}$ is a real Gaussian random vector with zero mean and an identity covariance matrix $K_{Z_{l}}=I$, for l∈{1,2}.

Both the noise variables, and the state variable are i.i.d. over channel uses. $G_{s_{1}}$ ($G_{s_{2}}$) is t×t real matrix that represents the channel matrix connecting the state source to the first (second) user. Similarly, $G_{1}$ ($G_{2}$) is a t×t real channel matrix connecting the helper to the first (second) user. Thus, our model captures a general scenario, where the helper’s power and the state power can be arbitrary.

Our goal is to characterize the capacity region of the Gaussian channel under various channel parameters $\left(G_{1},G_{2},G_{s_{1}},G_{s_{2}},K_{0},K_{1},K_{2},K_{S}\right)$.

### 3.2. Inner and Outer Bounds

In this section, we first derive inner and outer bounds on the capacity region for the state-dependent parallel channel with a helper. Then by comparing the inner and outer bounds, we characterize the segments on the capacity region boundary under various channel parameters.

We start by deriving an inner bound on the capacity region for the DMC based on the single-bin GP scheme.

**Proposition** **1.** *For the discrete memoryless state-dependent parallel channel with a helper under the same but differently scaled states at the two receivers, an inner bound on the capacity region consists of rate pairs $\left(R_{1},R_{2}\right)$ satisfying:*

(7a) $$R_{1}\leq \min\left\{I\left(W,X_{1};Y_{1}\right)-I\left(W;S\right),I\left(X_{1};Y_{1}|W\right)\right\},$$

(7b) $$R_{2}\leq \min\left\{I\left(W,X_{2};Y_{2}\right)-I\left(W;S\right),I\left(X_{2};Y_{2}|W\right)\right\},$$

*for some distribution $P_{W|S}P_{X_{0}|WS}P_{X_{1}}P_{X_{2}}$.*

**Proof.**  The proof is relegated to Appendix A. □

We evaluate the inner bound for the Gaussian channel by choosing the joint Gaussian distribution for random variables as follows:

(8) $$\begin{array}{c} W=X_{0}^{\prime }+AS,\\ X_{0}=X_{0}^{\prime }+BS,\\ X_{0}^{\prime }∼N\left(0,K_{0}^{\prime }\right),\\ X_{1}∼N\left(0,K_{1}\right)\;X_{2}∼N\left(0,K_{2}\right), \end{array}$$

where $X_{0}^{\prime },X_{1},X_{2},S$ are independent and $K_{0}^{\prime }⪯K_{0}$.

Let $f_{1}\left(\cdot \right),g_{1}\left(\cdot \right),f_{2}\left(\cdot \right)$ and $g_{2}\left(\cdot \right)$ be defined as

$$\begin{array}{cc} f_{1}\left(A,B,K_{0}^{\prime }\right) & =I\left(W,X_{1};Y_{1}\right)-I\left(W;S\right),\\ g_{1}\left(A,B,K_{0}^{\prime }\right) & =I(X_{1};Y_{1}|W),\\ f_{2}\left(A,B,K_{0}^{\prime }\right) & =I\left(W,X_{2};Y_{2}\right)-I\left(W;S\right),\\ g_{2}\left(A,B,K_{0}^{\prime }\right) & =I(X_{2};Y_{2}|W), \end{array}$$

where the mutual information terms are evaluated using the joint Gaussian distribution chosen in (8). Based on those definitions, we obtain an achievable region for the Gaussian channel.

**Proposition** **2.** *An inner bound on the capacity region of the parallel state-dependent MIMO Gaussian channel with same but differently scaled states and a state-cognitive helper consists of rate pairs $\left(R_{1},R_{2}\right)$ satisfying;*

(9a) $$R_{1}\leq \min\left\{f_{1}\left(A,B,K_{0}^{\prime }\right),g_{1}\left(A,B,K_{0}^{\prime }\right)\right\},$$

(9b) $$R_{2}\leq \min\left\{f_{2}\left(A,B,K_{0}^{\prime }\right),g_{2}\left(A,B,K_{0}^{\prime }\right)\right\},$$

*for some real matrices A, B and $K_{0}^{\prime }$ satisfying $K_{0}^{\prime }⪰0$, $K_{0}^{\prime }+BK_{S}B^{T}⪯K_{0}$.*

We note that the above choice of the helper’s signal incorporates two parts with $X_{0}^{\prime }$ designed using single-bin dirty paper coding, and BS acting as direct state subtraction.

We next present an outer bound which applies the point-to-point channel capacity and the upper bound derived for the point-to-point channel with a helper in [27].

Denote

(10) $$\begin{array}{c} R_{l}^{{\mathrm{ub}}_{1}}\left(\Sigma_{X_{0}S}\right)≜\frac{1}{2}\log\left(\frac{\left|G_{l}K_{0}G_{l}^{T}+K_{l}+G_{l}\Sigma_{X_{0}S}G_{s_{l}}+G_{s_{l}}\Sigma_{X_{0}S}^{T}G_{l}^{T}+G_{s_{l}}K_{S}G_{s_{l}}^{T}+I\right|}{\left|G_{l}K_{0}G_{l}^{T}+G_{l}\Sigma_{X_{0}S}G_{s_{l}}^{T}+G_{s_{l}}\Sigma_{X_{0}S}^{T}G_{l}^{T}+G_{s_{l}}K_{S}G_{s_{l}}^{T}+I\right|}\right)\\ +\frac{1}{2}\log\left(\left|G_{l}\left(K_{0}-\Sigma_{X_{0}S}K_{S}^{-1}\Sigma_{X_{0}S}^{T}\right)G_{l}^{T}+I\right|\right). \end{array}$$

**Proposition** **3.** *An outer bound on the capacity region of the state-dependent parallel MIMO Gaussian channel with a helper consists of rate pairs $\left(R_{1},R_{2}\right)$ satisfying:*

(11) $$R_{l}\leq \min\left\{R_{l}^{{\mathrm{ub}}_{1}}\left(\Sigma_{X_{0}S}\right),\frac{1}{2}\log\left(|K_{l}+I|\right)\right\},$$

*for every l∈{1,2} and $\Sigma_{X_{0}S}$ that satisfies $\Sigma_{X_{0}S}K_{S}^{-1}\Sigma_{X_{0}S}^{T}⪯K_{0}$.*

**Proof.**  The second term in (11) is simply the capacity of a point-to-point channel without state. The first term is derived in Appendix B. □

### 3.3. Capacity Region Characterization

In this section, we optimize *A* and *B* in Proposition 2, and compare the rate bounds with the outer bounds in Proposition 3 to characterize the points or segments on the capacity region boundary.

Since the inner bound in Proposition 2 is not convex, it is difficult to provide a closed form for the jointly optimized bounds. Therefore, we first optimize the bounds for $R_{1}$ and $R_{2}$ respectively, and then provide conditions on channel parameters such that these bounds match the outer bound. Based on the conditions, we partition the channel parameters into the sets, in which different segments of the capacity region boundary can be obtained.

We first consider the rate bound for $R_{1}$ in (9a). By setting

$$\begin{array}{c} A^{a}≜{\left(G_{1}K_{0}^{\prime }G_{1}^{T}+I\right)}^{-1}K_{0}^{\prime }G_{1}^{T}\left(G_{1}B+I\right),\;B^{a}≜\Sigma_{X_{0}S}^{☆}G_{s_{1}}K_{S}^{-1}, \end{array}$$

$f_{1}\left(A,B,K_{0}^{\prime }\right)$ takes the following form

$$\begin{array}{cc} f_{1}\left(A^{a},B^{a},K_{0}^{\prime }\right) & =\frac{1}{2}\log\left(\frac{|G_{1}K_{0}G_{1}^{T}+K_{1}+G_{1}\Sigma_{X_{0}S}^{☆}G_{s_{1}}+G_{s_{1}}^{T}\Sigma_{X_{0}S}^{☆T}G_{1}^{T}+G_{s_{1}}K_{S}G_{s_{1}}^{T}+I|}{|G_{1}K_{0}G_{1}^{T}+G_{1}\Sigma_{X_{0}S}^{☆}G_{s_{1}}+G_{s_{1}}^{T}\Sigma_{X_{0}S}^{☆T}G_{1}^{T}+G_{s_{1}}K_{S}G_{s_{1}}^{T}+I|}\right)\\ & \;+\frac{1}{2}\log\left(|G_{1}K_{0}^{\prime }G_{1}^{T}+I|\right), \end{array}$$

where $\Sigma_{X_{0}S}^{☆}$ maximizes $f_{1}\left(A^{a},B\left(\Sigma_{X_{0}S}\right),K_{0}^{\prime }\right)$. In fact, $A^{a}$ maximizes $f_{1}\left(A,B,K_{0}^{\prime }\right)$ for fixed *B*, and $B^{a}$ maximizes the function with $A=A^{a}$.

If $f_{1}\left(A^{a},B^{a},K_{0}^{\prime }\right)\leq g_{1}\left(A^{a},B^{a},K_{0}^{\prime }\right)$, $R_{1}=f_{1}\left(A^{a},B^{a},K_{0}^{\prime }\right)$ is achievable, and this matches the outer bound in (11). Thus, one segment of the capacity region is specified by

(12a) $$\begin{array}{c} R_{1}=f_{1}\left(A^{a},B^{a},K_{0}^{\prime }\right), \end{array}$$

(12b) $$\begin{array}{c} R_{2}\leq \min\left\{f_{2}\left(A^{a},B^{a},K_{0}^{\prime }\right),g_{2}\left(A^{a},B^{a},K_{0}^{\prime }\right)\right\}. \end{array}$$

We further observe that the second term $g_{1}\left(A,B,K_{0}^{\prime }\right)$ in (9a) is optimized by setting $A^{b}=B+G_{1}^{-1}G_{s_{1}}$, and hence

$$\begin{array}{c} g_{1}\left(A^{b},B,K_{0}^{\prime }\right)=\frac{1}{2}\log(|K_{1}+I\left|\right). \end{array}$$

If $g_{1}\left(B-G_{1}^{-1}G_{s_{1}},B,K_{0}^{\prime }\right)\leq f_{1}\left(B-G_{1}^{-1}G_{s_{1}},B,K_{0}^{\prime }\right)$, i.e.,

(13) $$K_{0}^{\prime }G_{1}K_{0}^{\prime }G_{1}^{T}⪰AK_{S}A^{T}\left(K_{1}+I\right)-K_{0}^{\prime }G_{1}AK_{S}A^{T}G_{1}^{T},$$

then the inner bound for $R_{1}$ becomes $R_{1}=\frac{1}{2}\log(|K_{1}+I\left|\right)$, which is the capacity of the point-to-point channel without state and matches the outer bound in (11). Thus, another segment of the capacity is specified by

(14a) $$\begin{array}{c} R_{1}=\frac{1}{2}\log(|K_{1}+I\left|\right), \end{array}$$

(14b) $$\begin{array}{c} R_{2}\leq \min\left\{f_{2}\left(A^{b},B,K_{0}^{\prime }\right),g_{2}\left(A^{b},B,K_{0}^{\prime }\right)\right\}. \end{array}$$

We then consider the rate bound for $R_{2}$. Similarly, the following segments on the capacity boundary can be obtained. If $f_{2}\left(A^{c},B^{c},K_{0}^{\prime }\right)\leq g_{2}\left(A^{c},B^{c},K_{0}^{\prime }\right)$, one segment of the capacity region boundary is specified by

(15a) $$\begin{array}{c} R_{1}\leq \min\left\{f_{1}\left(A^{c},B^{c},K_{0}^{\prime }\right),g_{1}\left(A^{c},B^{c},K_{0}^{\prime }\right)\right\}, \end{array}$$

(15b) $$\begin{array}{c} R_{2}=\frac{1}{2}\log\left(\frac{|G_{2}K_{0}G_{2}^{T}+K_{2}+G_{2}\Sigma_{X_{0}S}^{☆}G_{s_{2}}+G_{s_{2}}^{T}\Sigma_{X_{0}S}^{☆T}G_{2}^{T}+G_{s_{2}}K_{S}G_{s_{2}}^{T}+I|}{|G_{2}K_{0}G_{2}^{T}+G_{2}\Sigma_{X_{0}S}^{☆}G_{s_{2}}+G_{s_{2}}^{T}\Sigma_{X_{0}S}^{☆T}G_{2}^{T}+G_{s_{2}}K_{S}G_{s_{2}}^{T}+I|}\right)+\frac{1}{2}\log\left(|G_{2}K_{0}^{\prime }G_{2}^{T}+I|\right), \end{array}$$

where

$$A^{c}≜{\left(G_{2}K_{0}^{\prime }G_{2}^{T}+I\right)}^{-1}K_{0}^{\prime }G_{2}^{T}\left(G_{2}B+G_{S}\right),\;B^{c}≜\Sigma_{X_{0}S}^{☆☆}G_{s_{1}}K_{S}^{-1},$$

and $\Sigma_{X_{0}S}^{**}$ maximizes $f_{2}\left(A^{c},B^{c},K_{0}^{\prime }\right)$.

Furthermore, if $g_{2}\left(A,A-G_{2}^{-1}G_{s_{2}},K_{0}^{\prime }\right)\leq f_{2}\left(A,A-G_{2}^{-1}G_{s_{2}},K_{0}^{\prime }\right)$, one segment of the capacity region boundary is specified by

(16a) $$\begin{array}{c} R_{1}\leq \min\left\{f_{1}\left(A,A-G_{2}^{-1}G_{s_{2}},K_{0}^{\prime }\right),g_{1}\left(A,A-G_{2}^{-1}G_{s_{2}},K_{0}^{\prime }\right)\right\} \end{array}$$

(16b) $$\begin{array}{c} R_{2}=\frac{1}{2}\log(|K_{2}+I\left|\right). \end{array}$$

Appendix C describes how $\left(A^{a},B^{a}\right)$, $\left(A^{b},B^{b}\right)$, $\left(A^{c},B^{c}\right)$ and $\left(A^{d},B^{d}\right)$ were chosen.

Summarizing the above analysis, we obtain the following characterization of segments of the capacity region boundary.

**Theorem** **1.** *The channel parameters $\left(G_{1},G_{2},G_{s_{1}},G_{s_{2}},K_{0},K_{1},K_{2},K_{S}\right)$ can be partitioned into the sets $A_{1},B_{1},C_{1}$, where*

$$\begin{array}{cc} A_{1}= & \;\{\left(G_{1},G_{2},G_{s_{1}},G_{s_{2}},K_{0},K_{1},K_{2},K_{S}\right):f_{1}\left(A^{a},B^{a},K_{0}^{\prime }\right)\leq g_{1}\left(A^{a},B^{a},K_{0}^{\prime }\right)\},\\ C_{1}= & \;\{\left(G_{1},G_{2},G_{s_{1}},G_{s_{2}},K_{0},K_{1},K_{2},K_{S}\right):K_{0}^{\prime }G_{1}K_{0}^{\prime }G_{1}^{T}⪰AK_{S}A^{T}\left(K_{1}+I\right)-K_{0}^{\prime }G_{1}AK_{S}A^{T}G_{1}^{T}\\ & \;\;\mathrm{where}\;K_{0}^{\prime }=K_{0}-\left(A-G_{1}^{-1}G_{s_{1}}\right)K_{S}{\left(A-G_{1}^{-1}G_{s_{1}}\right)}^{T},\;\mathrm{for}\;\mathrm{some}\;A\in \Omega_{A}\},\\ B_{1}= & \;{\left(A_{1}\cup C_{1}\right)}^{c}. \end{array}$$

*If $\left(G_{1},G_{2},G_{s_{1}},G_{s_{2}},K_{0},K_{1},K_{2},K_{S}\right)\in A_{1}$, then (12a)–(12b) captures one segment of the capacity region boundary, where the state cannot be fully canceled. If $\left(G_{1},G_{2},G_{s_{1}},G_{s_{2}},K_{0},K_{1},K_{2},K_{S}\right)\in C_{1}$, then (14a)–(14b) captures one segment of the capacity region boundary where the state is fully canceled. If $\left(G_{1},G_{2},G_{s_{1}},G_{s_{2}},K_{0},K_{1},K_{2},K_{S}\right)\in B_{1}$, then the $R_{1}$ segment of the capacity region boundary is not characterized.*

*The channel parameters $\left(G_{1},G_{2},G_{s_{1}},G_{s_{2}},K_{0},K_{1},K_{2},K_{S}\right)$ can also be partitioned into the sets $A_{2},B_{2},C_{2}$, where*

$$\begin{array}{cc} A_{2} & =\{\left(G_{1},G_{2},G_{s_{1}},G_{s_{2}},K_{0},K_{1},K_{2},K_{S}\right):f_{2}\left(A^{c},B^{c},K_{0}^{\prime }\right)\leq g_{2}\left(A^{c},B^{c},K_{0}^{\prime }\right)\}\\ C_{2} & =\{\left(G_{1},G_{2},G_{s_{1}},G_{s_{2}},K_{0},K_{1},K_{2},K_{S}\right):K_{0}^{\prime }G_{2}K_{0}^{\prime }G_{2}^{T}⪰AK_{S}A^{T}\left(K_{2}+I\right)-K_{0}^{\prime }G_{2}AK_{S}A^{T}G_{2}^{T}\\ & \;\;\mathrm{where}\;K_{0}^{\prime }=K_{0}-\left(A-G_{2}^{-1}G_{s_{2}}\right)K_{S}{\left(A-G_{2}^{-1}G_{s_{2}}\right)}^{T},\;\mathrm{for}\;\mathrm{some}\;A\in \Omega_{A}\}\\ B_{2} & ={\left(A_{2}\cup C_{2}\right)}^{c}. \end{array}$$

*If $\left(G_{1},G_{2},G_{s_{1}},G_{s_{2}},K_{0},K_{1},K_{2},K_{S}\right)\in A_{2}$, then (15a)–(15b) captures one segment of the capacity region boundary, where the state cannot be fully canceled. If $\left(G_{1},G_{2},G_{s_{1}},G_{s_{2}},K_{0},K_{1},K_{2},K_{S}\right)\in C_{2}$, then (16a)–(16b) captures one segment of the capacity boundary where the state is fully canceled. If $\left(G_{1},G_{2},G_{s_{1}},G_{s_{2}},K_{0},K_{1},K_{2},K_{S}\right)\in B_{2}$, then the $R_{2}$ segment of the capacity region boundary is not characterized.*

The above theorem describes two partitions of the channel parameters, respectively under which segments on the capacity region boundary corresponding to $R_{1}$ and $R_{2}$ can be characterized. Intersection of two sets, each from one partition, collectively characterizes the entire segments on the capacity region boundary.

**Figure 6:**
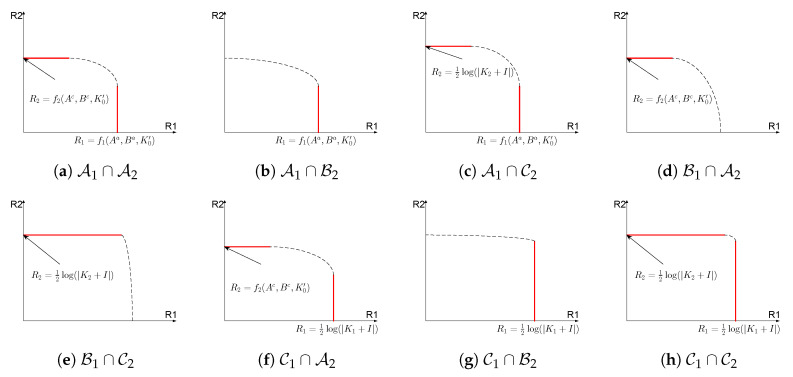
Segments of the capacity region for all cases of channel parameters.

Figure 6 lists all possible intersection of sets that the channel parameters can belong to. For each case in Figure 6, we use red solid line to represent the segments on the capacity region that are characterized in Theorem 1, and we also mark the value of the capacity that each segment corresponds to as characterized in Theorem 1. Please note that the case $B_{1}\cap B_{2}$ is not illustrated in Figure 6 since no segments are characterized in this case.

One interesting example in Theorem 1 is the case with $G_{1}^{-1}G_{s_{1}}=G_{2}^{-1}G_{s_{2}}$, in which $R_{1}$ and $R_{2}$ are optimized with the same set of coefficients *A* and *B* when $\left(G_{1},G_{2},G_{s_{1}},G_{s_{2}},K_{0},K_{1},K_{2},K_{S}\right)\in C_{1}\cap C_{2}$. Thus, the point-to-point channel capacity is simultaneously obtained for both $R_{1}$ and $R_{2}$, with state being fully canceled. We state this result in the following theorem.

**Theorem** **2.** *If $G_{1}^{-1}G_{s_{1}}=G_{2}^{-1}G_{s_{2}}$, $K_{0}^{\prime }G_{1}K_{0}^{\prime }G_{1}^{T}⪰AK_{S}A^{T}\left(K_{1}+I\right)-K_{0}^{\prime }G_{1}AK_{S}A^{T}G_{1}^{T}$ and $K_{0}^{\prime }G_{2}K_{0}^{\prime }G_{2}^{T}⪰AK_{S}A^{T}\left(K_{2}+I\right)-K_{0}^{\prime }G_{2}AK_{S}A^{T}G_{2}^{T}$ where $K_{0}^{\prime }=K_{0}-\left(A-G_{1}^{-1}G_{s_{1}}\right)K_{S}{\left(A-G_{1}^{-1}G_{s_{1}}\right)}^{T}$, for some $A\in \Omega_{A}$ then the capacity region of the state-dependent parallel Gaussian channel with a helper and under the same but differently scaled states contains $\left(R_{1},R_{2}\right)$ satisfying*

$$\begin{array}{cc} R_{1}\leq & \;0.5\log(|K_{1}+I|),\\ R_{2}\leq & \;0.5\log(|K_{2}+I|). \end{array}$$

The channel conditions of Theorem 2 are not just of mathematical importance but also have a practical utility. Consider, for example, a scenario where the helper is also the interferer (see Figure 3), in such case it is reasonable to assume that $G_{s_{1}}=G_{1}$ and $G_{s_{2}}=G_{2}$, and thus the aforementioned conditions are satisfied.

### 3.4. Numerical Example

We now examine our results via simulations. In particular, we focus on the scalar channel case, i.e., $G_{1}\leftarrow 1$, $G_{2}\leftarrow b$, $G_{s_{1}}\leftarrow 1$, $G_{s_{2}}\leftarrow a$, $K_{0}\leftarrow P_{0}$, $K_{0}^{\prime }\leftarrow P_{0}^{\prime }$, $K_{1}\leftarrow P_{1}$, $K_{2}\leftarrow P_{2}$ and $K_{S}\leftarrow Q$. Furthermore, we denote A←α, B←β and $\rho_{0S}≜\beta \sqrt{P_{0}Q}$.

**Figure 7:**
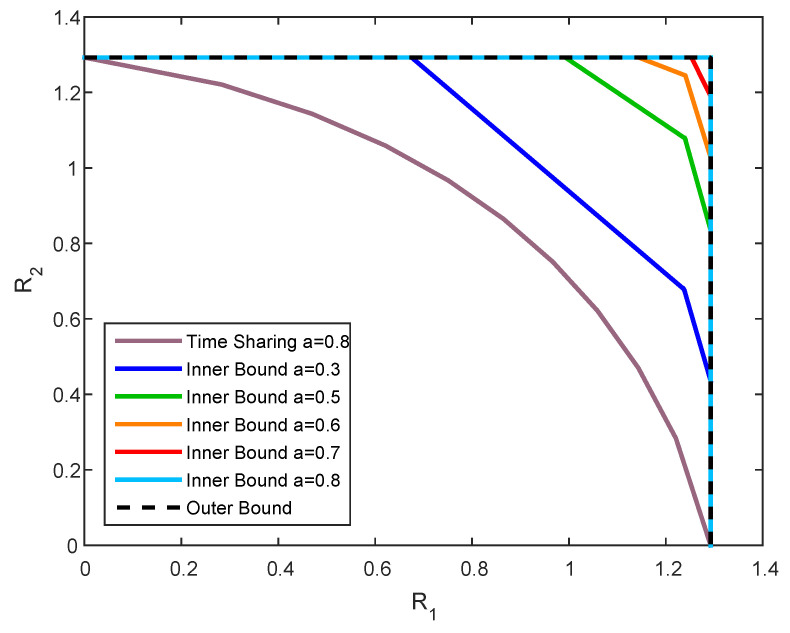
Capacity bounds for channel parameters $P_{0}=6$, $P_{1}=P_{2}=5$, Q=12, b=0.8 and various state gain *a*.

We set $P_{0}=6$, $P_{1}=P_{2}=5$, Q=12, and b=0.8, and plot the inner and outer bounds for the capacity region $\left(R_{1},R_{2}\right)$ for two values of *a*. It can be observed from Figure 7 that the upper bound is defined by the rectangular region of channel without state. The inner bound, in the contrary, is susceptible to the value of *a*, such that in the case where a=b, our inner and outer bounds coincide everywhere, while in the case a≠b they coincide only on some segments. Both observations corroborate the characterization of the capacity in Theorems 1 and 2.

It is also interesting to illustrate how the channel parameters (a,b) affect our ability to characterize the capacity region boundary. For this we propose the following setup:

- we choose α and β such that $R_{1}$ lies on the capacity region boundary;
- we further choose $\rho_{0S}$ that maximizes the achievable $R_{2}$, denoted as $R_{2}^{I}$;
- we compare it to the outer bound of $R_{2}$, $R_{2}^{O}$, and plot the gap $\Delta ≜R_{2}^{O}-R_{2}^{I}$.

**Figure 8:**
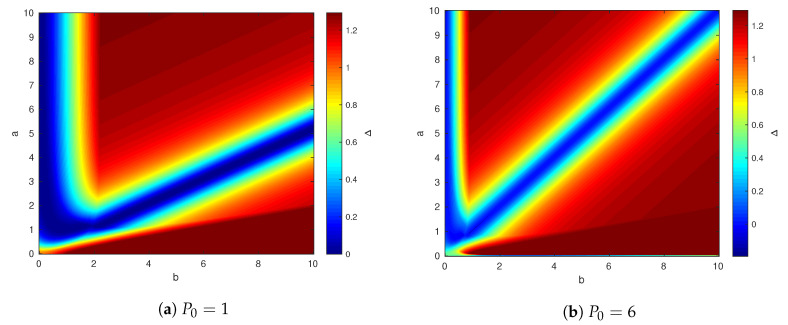
Capacity gap for foxed $P_{0}$.

Figure 8 shows the results of such simulation for two values of $P_{0}$: $P_{0}=1$ for which the state is not fully canceled for user 1 and $P_{0}=6$, for which the state is canceled. We fix other parameters as before, that is $P_{1}=P_{2}=5$ and Q=12. The right figure shows that the capacity gap is small around the line a=b, this result is not surprising, and it appears in Theorem 2. The left Figure is also interesting. It shows that there is a curve a≠b for which the capacity gap is also near zero. The reason for this phenomenon is explained as follows.

- The chosen channel parameters satisfy $\left(a,b,P_{0},P_{1},P_{2},Q\right)\in A_{1}$, and hence
  (17) $$\alpha_{1}=\frac{\left(1+\beta_{1}\right)P_{0}^{\prime }}{P_{0}^{\prime }+1}\;\beta_{1}=\rho_{0S}^{☆}\sqrt{\frac{P_{0}}{Q}}$$
  optimize $R_{1}$.
- Thus, if $\frac{a}{b}$ satisfies
  (18) $$\frac{a}{b}=\alpha_{1}-\beta_{1},$$
  and $b^{2}P_{0}^{\prime 2}\geq \alpha_{1}^{2}Q\left(P_{2}+1-b^{2}P_{0}^{\prime }\right)$, then $\left(a,b,P_{0},P_{1},P_{2},Q\right)\in C_{2}$, i.e., $R_{2}=\frac{1}{2}\log\left(1+P_{2}\right)$ is achievable.

**Figure 9:**
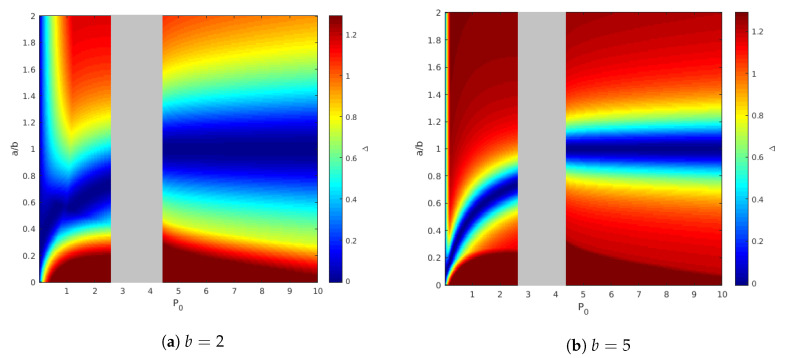
Capacity gap for fixed *b*.

We illustrate this result in Figure 9, where we fixed the channel parameters b=1, $P_{1}=P_{2}=5$, Q=12, and calculate the capacity gap for various values of *a* and $P_{0}$. The shaded area is the region of $P_{0}$ where the capacity of the point-to-point helper channel is not characterized.

**Figure 10:**
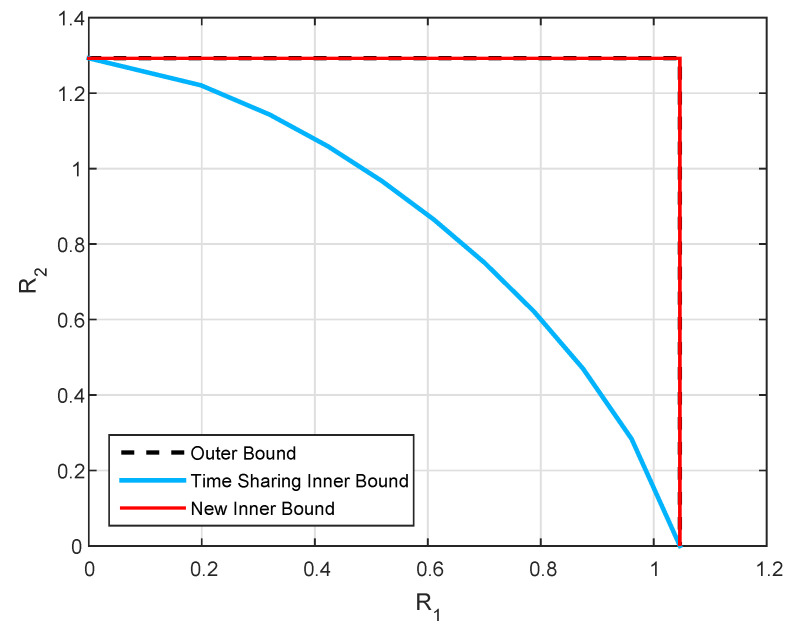
Inner and outer bounds for $\left(a,b,P_{0},P_{1},P_{2},Q\right)=\left(3.5,5,2,5,5,12\right)$.

In practical situations the channel parameters *a* and *b* are fixed but the helper can control $P_{0}$. The results here imply that for a fixed (a,b) we can choose $P_{0}$ such that the capacity gap is close to zero. We emphasize this in Figure 10, where we plot the inner and outer bounds on achievable $\left(R_{1},R_{2}\right)$ with the following channel parameters

(19) $$\left(a,b,P_{0},P_{1},P_{2},Q\right)=\left(3.5,5,2.17,5,5,12\right).$$

## 4. MIMO Gaussian Channel with Independent States

In this section, we consider the problem of channel coding over MIMO Gaussian parallel state-dependent channel with a cognitive helper where the states are independent. We start with deriving an achievable region for a general discrete memoryless case. We then, evaluate this region for the Gaussian setting by choosing an appropriate jointly Gaussian input distribution.

### 4.1. Problem Formulation

**Figure 11:**
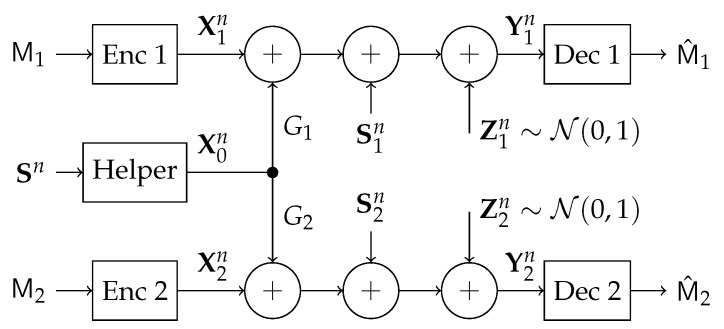
MIMO State-Dependent Parallel Channel with a Helper.

Consider a 3-transmitter, 2-receiver *state-dependent parallel DMC* depicted in Figure 11, where Transmitter 1 wishes to communicate a message $M_{1}$ to Receiver 1, and similarly Transmitter 2 wishes to transmit a message $M_{2}$ to its corresponding Receiver 2. The messages $M_{1}$ and $M_{2}$ are independent. The communication takes over a parallel state-dependent channel characterized by a probability transition matrix $p(y_{1},y_{2}|x_{0},x_{1},x_{2},s)$. The transmitter at the helper has non-causal knowledge of the state and tries to mitigate the interference caused in both channels. The state variable S is random taking values in S and drawn from a discrete memoryless source (DMS)

$$\begin{array}{c} P_{S^{n}}\left(s^{n}\right)=\prod_{i=1}^{n}P_{S}\left(s_{i}\right). \end{array}$$

A $\left(2^{nR_{1}},2^{nR_{2}},n\right)$ code for the parallel state-dependent channel with state known non-causally at the helper consists of

- two message sets $I_{R_{1}}^{\left(\;n\;\right)}$ and $I_{R_{2}}^{\left(\;n\;\right)}$,
- three encoders, where the encoder at the helper assigns a codeword $x_{0}^{n}\left(s^{n}\right)$ to each state sequence $s^{n}\in S^{n}$, encoder 1 assigns a codeword $x_{1}^{n}\left(m_{1}\right)$ to each message $m_{1}\in I_{R_{1}}^{\left(\;n\;\right)}$ and encoder 2 assigns a codeword $x_{2}^{n}\left(m_{2}\right)$ to each message $m_{2}\in I_{R_{2}}^{\left(\;n\;\right)}$, and
- two decoders, where decoder 1 assigns an estimate ${\hat{m}}_{1}\in I_{R_{1}}^{\left(\;n\;\right)}$ or an error message e to each received sequence $y_{1}^{n}$, and decoder 2 assigns an estimate $\hat{m_{2}}\in I_{R_{2}}^{\left(\;n\;\right)}$ or an error message e to each received sequence $y_{2}^{n}$.

We assume that the message pair $\left(M_{1},M_{2}\right)$ is uniformly distributed over $I_{R_{1}}^{\left(\;n\;\right)}\times I_{R_{2}}^{\left(\;n\;\right)}$. The average probability of error for a length-*n* code is defined as

(20) $$P_{e}^{\left(n\right)}=P\left\{{\hat{M}}_{1}\neq M_{1}\;\mathrm{or}\;{\hat{M}}_{2}\neq M_{2}\right\}.$$

A rate pair $\left(R_{1},R_{2}\right)$ is said to be achievable if there exists a sequence of $\left(2^{nR_{1}},2^{nR_{2}},n\right)$ codes such that $\lim_{n\to \infty }P_{e}^{\left(n\right)}=0$. The capacity region C is the closure of the set of all achievable rate pairs $\left(R_{1},R_{2}\right)$.

We observe that due to the lack of cooperation between the receivers, the capacity region of this channel depends on the $p(y_{1},y_{2}|x_{0},x_{1},x_{2},s)$ only through the conditional marginal PMFs $p(y_{1}|x_{0},x_{1},s)$ and $p(y_{2}|x_{0},x_{2},s)$. This observation is similar to the DM-BC ([37], Lemma 5.1).

Our goal is to characterize the capacity region C for the state-dependent Gaussian parallel channel with additive state known at the helper. Here, the state $S={\left(S_{1},S_{2}\right)}^{T}$. The channel is modeled by a Gaussian vector parallel state-dependent channel

(21) $$Y_{l}=G_{l}X_{0}+X_{l}+S_{l}+Z_{l},\;l=1,2,$$

where $G_{1}$, $G_{2}$ are t×t channel gain matrices. $X_{0}$, $X_{1}$, $X_{2}$ are the helper and the noncognitive transmitters channel input signals, each subject to an average matrix power constraint

(22) $$\frac{1}{n}\sum_{i=1}^{n}X_{l,i}X_{l,i}^{T}⪯K_{l},\;l=0,1,2.$$

The additive state variables $S_{l}$ and noise $Z_{l}$ are independent and identically distributed (i.i.d.) Gaussian with zero mean and strictly positive definite covariance matrix $K_{S_{l}}$ and *I* respectively.

### 4.2. Outer and Inner Bounds

To characterize the capacity region of this channel, we first consider the following outer bound on the capacity region for the Gaussian setting.

Let,

(23) $$K_{S}≜\left(\begin{array}{cc} K_{S_{1}} & 0\\ 0 & K_{S_{2}} \end{array}\right),$$

and

(24) $$\begin{array}{c} R_{l}^{{\mathrm{ub}}_{2}}\left(\Sigma_{X_{0}S}\right)≜\frac{1}{2}\log\left(\frac{|G_{l}K_{0}G_{l}^{T}+K_{l}+G_{l}\Sigma_{X_{0}S_{l}}+\Sigma_{X_{0}S_{l}}^{T}G_{l}^{T}+K_{S_{l}}+I|}{|G_{l}K_{0}G_{l}^{T}+G_{l}\Sigma_{X_{0}S_{l}}+\Sigma_{X_{0}S_{l}}^{T}G_{l}^{T}+K_{S_{l}}+I|}\right)\\ +\frac{1}{2}\log\left(|G_{l}\left(K_{0}-\Sigma_{X_{0}S}K_{S}^{-1}\Sigma_{X_{0}S}^{T}\right)G_{l}^{T}+I|\right). \end{array}$$

**Proposition** **4.** *Every achievable rate pair $\left(R_{1},R_{2}\right)$ of the state-dependent parallel Gaussian channel with a helper must satisfy the following inequalities*

(25) $$R_{l}\leq \min\left\{R_{l}^{{\mathrm{ub}}_{2}}\left(\Sigma_{X_{0}S}\right),\frac{1}{2}\log(|K_{l}+I\left|\right)\right\},$$

*for l={1,2} and some covariance matrices $\left(\Sigma_{XS_{1}},\Sigma_{XS_{2}}\right)$, such that $\Sigma_{X_{0}S}K_{S}^{-1}\Sigma_{X_{0}S}^{T}⪯K_{0}$, where*

(26) $$\Sigma_{X_{0}S}≜\left(\begin{array}{cc} \Sigma_{X_{0}S_{1}} & \Sigma_{X_{0}S_{2}} \end{array}\right).$$

The proof of this outer bound is quite similar to the proof of the outer bound in Proposition 3 and is given in Appendix D.

The upper bound for each rate consists of two terms, the first one reflects the scenario when the interference cannot be completely canceled, and the second is simply the point-to-point capacity of the channel without the state. Furthermore, the individual rate bounds are connected through the choice of $\Sigma_{X_{0}S_{1}}$ and $\Sigma_{X_{0}S_{2}}$.

We next derive an achievable region for the channel based on an achievable scheme that integrates Marton’s coding, single-bin dirty paper coding, and state cancelation. More specifically, we generate two auxiliary random variables, U and V to incorporate the state information so that Receiver 1 (and respectively 2) decodes U (and respectively V) and then decodes the respective transmitter information. Based on such an achievable scheme, we derive the following inner bound on the capacity region for the DM case.

**Proposition** **5.** *An inner bound on the capacity region of the discrete memoryless parallel state-dependent channel with a helper consists of rate pairs $\left(R_{1},R_{2}\right)$ satisfying:*

(27a) $$R_{1}\leq \min\left\{I\left(U,X_{1};Y_{1}\right)-I\left(U;S\right),I\left(X_{1};Y_{1}|U\right)\right\},$$

(27b) $$R_{2}\leq \min\left\{I\left(V,X_{2};Y_{2}\right)-I\left(V;S\right),I\left(X_{2};Y_{2}|V\right)\right\},$$

(27c) $$\begin{array}{c} R_{1}+R_{2}\leq \min\{I\left(U,X_{1};Y_{1}\right)-I\left(U;S\right)+I\left(V,X_{2};Y_{2}\right)-I\left(V;S\right)-I\left(V;U|S\right),\\ I\left(X_{1};Y_{1}|U\right)+I\left(X_{2};Y_{2}|V\right)\}, \end{array}$$

*for some PMF $P_{UVX_{0}|S}P_{X_{1}}P_{X_{2}}$.*

**Remark** **1.** *The achievable region in Proposition 5 is equivalent to the following region*

(28a) $$\begin{array}{cc} R_{1} & \leq \min\{I\left(U,X_{1};Y_{1}\right)-I\left(U;S\right),I\left(X_{1};Y_{1}|U\right)\}, \end{array}$$

(28b) $$\begin{array}{cc} R_{2} & \leq \min\{I\left(V,X_{2};Y_{2}\right)-I\left(V;U,S\right),I\left(X_{2};Y_{2}|V\right)\}, \end{array}$$

*for some PMF $P_{UVX_{0}|S}P_{X_{1}}P_{X_{2}}$.*

**Proof.**  The proof of the inner bound is relegated to Appendix E. □

We evaluate the latter inner bound for the Gaussian channel by choosing the joint Gaussian distribution for random variables as follows:

(29) $$\begin{array}{c} U=X_{01}^{\prime }+A_{11}S_{1}+A_{12}S_{2},\\ V=X_{02}^{\prime }+A_{20}X_{01}^{\prime }+A_{21}S_{1}+A_{22}S_{2},\\ X_{0}=X_{01}^{\prime }+B_{1}S_{1}+X_{02}^{\prime }+B_{2}S_{2},\\ X_{01}^{\prime }∼N\left(0,K_{01}^{\prime }\right)\;X_{02}^{\prime }∼N\left(0,K_{02}^{\prime }\right),\\ X_{1}∼N\left(0,K_{1}\right)\;X_{2}∼N\left(0,K_{2}\right), \end{array}$$

where $X_{01}^{\prime },X_{02}^{\prime },X_{1},X_{2},S_{1},S_{2}$ are independent. For simplicity of representation, denote ${\bar{A}}_{1}=\left(A_{11},A_{12}\right)$, ${\bar{A}}_{2}=\left(A_{20},A_{11},A_{12}\right)$ and $\bar{B}=\left(B_{1},B_{2}\right)$. Let $f_{1}\left(\cdot \right),g_{1}\left(\cdot \right),f_{2}\left(\cdot \right)$ and $g_{2}\left(\cdot \right)$ be defined as

$$\begin{array}{cc} f_{1}\left({\bar{A}}_{1},\bar{B},K_{01}^{\prime },K_{02}^{\prime }\right) & =I\left(U,X_{1};Y_{1}\right)-I\left(U;S\right),\\ g_{1}\left({\bar{A}}_{1},\bar{B},K_{01}^{\prime },K_{02}^{\prime }\right) & =I(X_{1};Y_{1}|U),\\ f_{2}\left({\bar{A}}_{2},\bar{B},K_{01}^{\prime },K_{02}^{\prime }\right) & =I\left(V,X_{2};Y_{2}\right)-I\left(V;U,S\right),\\ g_{2}\left({\bar{A}}_{2},\bar{B},K_{01}^{\prime },K_{02}^{\prime }\right) & =I(X_{2};Y_{2}|V), \end{array}$$

where the mutual information terms are evaluated using the joint Gaussian distribution set at (29). Based on those definitions we obtain an achievable region for the Gaussian channel.

**Proposition** **6.** *An inner bound on the capacity region of the parallel state-dependent Gaussian channel with a helper and with independent states, consists of rate pairs $\left(R_{1},R_{2}\right)$ satisfying;*

(30a) $$R_{1}\leq \min\left\{f_{1}\left({\bar{A}}_{1},\bar{B},K_{01}^{\prime },K_{02}^{\prime }\right),g_{1}\left({\bar{A}}_{1},\bar{B},K_{01}^{\prime },K_{02}^{\prime }\right)\right\},$$

(30b) $$R_{2}\leq \min\left\{f_{2}\left({\bar{A}}_{2},\bar{B},K_{01}^{\prime },K_{02}^{\prime }\right),g_{2}\left({\bar{A}}_{2},\bar{B},K_{01}^{\prime },K_{02}^{\prime }\right)\right\},$$

*for some real matrices $A_{20}$, $A_{21}$, $A_{22}$, $B_{1}$, $B_{2}$, $K_{01}^{\prime }$ and $K_{02}^{\prime }$ satisfying $K_{01}^{\prime },K_{02}^{\prime }⪰0$, $K_{01}^{\prime }+K_{02}^{\prime }+B_{1}K_{S_{1}}B_{1}^{T}+B_{2}K_{S_{2}}B_{2}^{T}⪯K_{0}$.*

Now we provide our intuition behind such construction of the RVs in the proof of Proposition 6. $X_{0}$ contains two parts, the one with $B_{l}$, l=1,2 controls the direct state cancelation of each state. The second part $X_{0l}^{\prime }$, l=1,2, is used for dirty paper coding via generation of the state-correlated auxiliary RVs U and V.

### 4.3. Capacity Region Characterization

In this section, we will characterize segments on the capacity boundary for various channel parameters using the inner and outer bounds that were derived in Section 4.2. Consider the inner bounds in (30a)–(30b). Each bound has two terms in the argument of min. We suggest optimizing each term independently and then comparing it to the outer bounds in (25). In the last step we will state the conditions under which those terms are valid. Our technique for optimal choice of $\left(A_{11},,A_{12},A_{20},A_{21},A_{22}\right)$ be such that cancels the respective interfering terms from the mutual information quantities. We explain how those matrices were chosen in Appendix F.

We begin by considering what choice of $\left(A_{11},A_{12}\right)$ can maximize $f_{1}\left({\bar{A}}_{1},\bar{B},K_{01}^{\prime },K_{02}^{\prime }\right)$. Let

$$\begin{array}{cc} A_{11}^{a} & ={\left(G_{1}\left(K_{01}^{\prime }+K_{02}^{\prime }\right)G_{1}^{T}+I\right)}^{-1}K_{01}^{\prime }G_{1}^{T}\left(G_{1}B_{1}+I\right),\\ A_{12}^{a} & ={\left(G_{1}\left(K_{01}^{\prime }+K_{02}^{\prime }\right)G_{1}^{T}+I\right)}^{-1}K_{01}^{\prime }G_{1}^{T}G_{1}B_{2}. \end{array}$$

Then $f_{1}\left({\bar{A}}_{1},\bar{B},K_{01}^{\prime },K_{02}^{\prime }\right)$ takes the following form

(31) $$\begin{array}{c} f_{1}\left({\bar{A}}_{1}^{a},\bar{B},K_{01}^{\prime },K_{02}^{\prime }\right)=\frac{1}{2}\log\left(\frac{|G_{1}K_{0}G_{1}^{T}+K_{1}+G_{1}B_{1}K_{S_{1}}+K_{S_{1}}B_{1}^{T}G_{1}^{T}+K_{S_{1}}+I|}{|G_{1}K_{0}G_{1}^{T}+G_{1}B_{1}K_{S_{1}}+K_{S_{1}}B_{1}^{T}G_{1}^{T}+I|}\right)\\ +\frac{1}{2}\log\left(\frac{|G_{1}\left(K_{01}^{\prime }+K_{02}^{\prime }\right)G_{1}^{T}+I|}{|G_{1}K_{02}^{\prime }G_{1}^{T}+I|}\right). \end{array}$$

If $f_{1}\left({\bar{A}}_{1}^{a},{\bar{B}}^{a},K_{01}^{\prime },K_{02}^{\prime }\right)\leq g_{1}\left({\bar{A}}_{1}^{a},{\bar{B}}^{a},K_{01}^{\prime },K_{02}^{\prime }\right)$, then $R_{1}=f_{1}\left({\bar{A}}_{1}^{a},{\bar{B}}^{a},K_{01}^{\prime },K_{02}^{\prime }\right)$ is achievable. Moreover, if we choose $K_{02}^{\prime }=0$, then $R_{1}=f_{1}\left(A_{11}^{a},A_{12}^{a},B_{1}^{a},B_{2}^{a},K_{0}^{\prime },0\right)$ meets the outer bound (the first term in “min” in (25)) with $B_{1}K_{S_{1}}=\Sigma_{X_{0}S_{1}}$ and $B_{2}K_{S_{2}}=\Sigma_{X_{0}S_{2}}$. Furthermore, by setting

$$\begin{array}{c} A_{11}^{b}=B_{1}+G_{1}^{-1},\;A_{12}^{b}=B_{2}, \end{array}$$

we obtain

$$\begin{array}{c} g_{1}\left({\bar{A}}_{1}^{b},\bar{B},K_{01}^{\prime },K_{02}^{\prime }\right)=\frac{1}{2}\log\left(\frac{|G_{1}K_{02}^{\prime }G_{1}^{T}+K_{1}+I|}{|G_{1}K_{02}^{\prime }G_{1}^{T}+I|}\right). \end{array}$$

If $g_{1}\left({\bar{A}}_{1}^{b},\bar{B},K_{01}^{\prime },K_{02}^{\prime }\right)\leq f_{1}\left({\bar{A}}_{1}^{b},\bar{B},K_{01}^{\prime },K_{02}^{\prime }\right)$, then

$$\begin{array}{c} R_{1}=\frac{1}{2}\log\left(\frac{|G_{1}K_{02}^{\prime }G_{1}^{T}+K_{1}+I|}{|G_{1}K_{02}^{\prime }G_{1}^{T}+I|}\right) \end{array}$$

is achievable. Similarly, by choosing $K_{02}^{\prime }=0$, then $R_{1}=\frac{1}{2}\log\left|K_{1}+I\right|$ is achievable and this meets the outer bound (the second term in “min” in (25)). Next we consider the bound on $R_{2}$. Let

$$\begin{array}{cc} A_{20}^{a} & ={\left(G_{2}K_{02}^{\prime }G_{2}^{T}+I\right)}^{-1}K_{02}^{\prime }G_{2}^{T}G_{2},\\ A_{21}^{a} & ={\left(G_{2}K_{02}^{\prime }G_{2}^{T}+I\right)}^{-1}K_{02}^{\prime }G_{2}^{T}G_{2}B_{1},\\ A_{22}^{a} & ={\left(G_{2}K_{02}^{\prime }G_{2}^{T}+I\right)}^{-1}K_{02}^{\prime }G_{2}^{T}\left(G_{2}B_{2}+I\right). \end{array}$$

Then $f_{2}\left({\bar{A}}_{2},\bar{B},K_{01}^{\prime },K_{02}^{\prime }\right)$ takes the following form

(32) $$\begin{array}{c} f_{2}\left({\bar{A}}_{2}^{a},\bar{B},K_{01}^{\prime },K_{02}^{\prime }\right)=\frac{1}{2}\log\left(\frac{|G_{2}K_{0}G_{2}^{T}+K_{2}+G_{2}B_{2}K_{S_{2}}+K_{S_{2}}B_{2}^{T}G_{2}^{T}+K_{S_{2}}+I|}{|G_{2}K_{0}G_{2}^{T}+G_{2}B_{2}K_{S_{2}}+K_{S_{2}}B_{2}^{T}G_{2}^{T}+K_{S_{2}}+I|}\right)\\ +\frac{1}{2}\log\left(|G_{2}K_{02}^{\prime }G_{2}^{T}+I|\right). \end{array}$$

If $f_{2}\left({\bar{A}}_{2}^{a},\bar{B},K_{01}^{\prime },K_{02}^{\prime }\right)\leq g_{2}\left({\bar{A}}_{2}^{a},\bar{B},K_{01}^{\prime },K_{02}^{\prime }\right)$, then $R_{2}=f_{2}\left({\bar{A}}_{2}^{a},\bar{B},K_{01}^{\prime },K_{02}^{\prime }\right)$ is achievable. Moreover, if we choose $K_{01}^{\prime }=0$, then $R_{2}=f_{2}\left({\bar{A}}_{2}^{a},\bar{B},0,K_{0}^{\prime }\right)$ meets the outer bound (the first term in “min” in (25)).

Furthermore, we set

(33) $$A_{20}^{b}=I,\;A_{21}^{b}=B_{1},\;A_{22}^{b}=B_{2}+G_{2}^{-1},$$

and then obtain

(34) $$g_{2}\left({\bar{A}}_{2}^{b},\bar{B},K_{01}^{\prime },K_{02}^{\prime }\right)=\frac{1}{2}\log\left(|K_{2}+I|\right).$$

If $g_{2}\left({\bar{A}}_{2}^{b},\bar{B},K_{01}^{\prime },K_{02}^{\prime }\right)\leq f_{2}\left({\bar{A}}_{2}^{b},\bar{B},K_{01}^{\prime },K_{02}^{\prime }\right)$, then $R_{2}=\frac{1}{2}\log\left(|K_{2}+I|\right)$ is achievable and this meets the outer bound. This also equals the maximum rate for $R_{2}$ when the channel is not corrupted by state.

Summarizing the above analysis, we obtain the following characterization of segments of the capacity region boundary.

**Theorem** **3.** *The channel parameters $\left(G_{1},G_{2},K_{0},K_{1},K_{2},K_{S_{1}},K_{S_{2}}\right)$ can be partitioned into the sets $A_{1},B_{1},C_{1}$, where*

$$\begin{array}{cc} A_{1}= & \;\{\left(G_{1},G_{2},K_{0},K_{1},K_{2},K_{S_{1}},K_{S_{2}}\right):f_{1}\left({\bar{A}}_{1}^{a},{\bar{B}}^{a},K_{01}^{\prime },K_{02}^{\prime }\right)\leq g_{1}\left({\bar{A}}_{1}^{a},{\bar{B}}^{a},K_{01}^{\prime },K_{02}^{\prime }\right),\\ C_{1}= & \;\{\left(G_{1},G_{2},K_{0},K_{1},K_{2},K_{S_{1}},K_{S_{2}}\right):f_{1}\left({\bar{A}}_{1}^{b},\bar{B},K_{01}^{\prime },K_{02}^{\prime }\right)\geq g_{1}\left({\bar{A}}_{1}^{b},\bar{B},K_{01}^{\prime },K_{02}^{\prime }\right)\},\\ B_{1}= & \;{\left(A_{1}\cup C_{1}\right)}^{c}. \end{array}$$

*If $\left(G_{1},G_{2},K_{0},K_{1},K_{2},K_{S_{1}},K_{S_{2}}\right)\in A_{1}$, then $R_{1}=f_{1}\left({\bar{A}}_{1}^{a},\bar{B},K_{0}^{\prime },0\right)$ captures one segment of the capacity region boundary, where the state cannot be fully canceled. If $\left(G_{1},G_{2},K_{0},K_{1},K_{2},K_{S_{1}},K_{S_{2}}\right)\in C_{1}$, then $R_{1}=\frac{1}{2}\log\left|K_{1}+I\right|$ captures one segment of the capacity region boundary where the state is fully canceled. If $\left(G_{1},G_{2},K_{0},K_{1},K_{2},K_{S_{1}},K_{S_{2}}\right)\in B_{1}$, then the $R_{1}$ segment of the capacity region boundary is not characterized.*

*The channel parameters $\left(G_{1},G_{2},K_{0},K_{1},K_{2},K_{S_{1}},K_{S_{2}}\right)$ can also be partitioned into the sets $A_{2},B_{2},C_{2}$, where*

$$\begin{array}{cc} A_{2}= & \;\{\left(G_{1},G_{2},K_{0},K_{1},K_{2},K_{S_{1}},K_{S_{2}}\right):f_{2}\left({\bar{A}}_{2}^{a},\bar{B},K_{01}^{\prime },K_{02}^{\prime }\right)\leq g_{2}\left({\bar{A}}_{2}^{a},\bar{B},K_{01}^{\prime },K_{02}^{\prime }\right),\\ C_{2}= & \;\{\left(G_{1},G_{2},K_{0},K_{1},K_{2},K_{S_{1}},K_{S_{2}}\right):f_{2}\left({\bar{A}}_{2}^{b},\bar{B},K_{01}^{\prime },K_{02}^{\prime }\right)\geq g_{2}\left({\bar{A}}_{2}^{b},\bar{B},K_{01}^{\prime },K_{02}^{\prime }\right),\\ B_{2}= & \;{\left(A_{2}\cup C_{2}\right)}^{c}. \end{array}$$

*If $\left(G_{1},G_{2},K_{0},K_{1},K_{2},K_{S_{1}},K_{S_{2}}\right)\in A_{2}$, then $R_{2}=f_{2}\left({\bar{A}}_{2}^{a},\bar{B},0,K_{0}^{\prime }\right)$ captures one segment of the capacity region boundary, where the state cannot be fully canceled. If $\left(G_{1},G_{2},K_{0},K_{1},K_{2},K_{S_{1}},K_{S_{2}}\right)\in C_{2}$, then $R_{2}=\frac{1}{2}\log\left(|K_{2}+I|\right)$ captures one segment of the capacity boundary where the state is fully canceled. If $\left(G_{1},G_{2},K_{0},K_{1},K_{2},K_{S_{1}},K_{S_{2}}\right)\in B_{2}$, then the $R_{2}$ segment of the capacity region boundary is not characterized.*

The above theorem describes two partitions of the channel parameters, respectively under which segments on the capacity region boundary corresponding to $R_{1}$ and $R_{2}$ can be characterized. Intersection of two sets, each from one partition, collectively characterizes the entire segments on the capacity region boundary.

We note that our inner bound can be tight for some set of channel parameters. As an example, assume that $\left(G_{1},G_{2},K_{0},K_{1},K_{2},K_{S_{1}},K_{S_{2}}\right)\in C_{1}\cap C_{2}$. In such case, $R_{1}=\frac{1}{2}\log\left(\frac{|G_{1}K_{02}^{\prime }G_{1}^{T}+K_{1}+I|}{|G_{1}K_{02}^{\prime }G_{1}^{T}+I|}\right)$ and $R_{2}=\frac{1}{2}\log\left(|K_{2}+I|\right)$ are achievable. For the point-to-point helper channel [28], it was shown that if the helper power is above some threshold, the state is completely canceled, whereas in our model we have two parallel channels. If the helper power is high enough, it can split its signal, similarly as for the Gaussian BC, such that one part of it is intended for Receiver 2, where by using dirty paper coding it eliminates completely the interference caused by the state and the part of the signal intended for Receiver 1. In the same time the part of the helper signal intended for Receiver 1, can only cancel the interference caused by the state while the part intended to Receiver 2 is treated as noise.

### 4.4. Numerical Results

**Figure 12:**
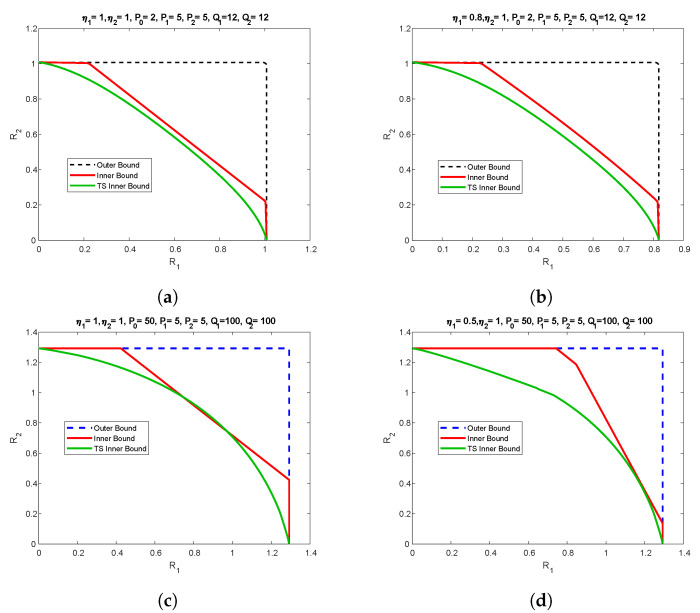
Numerical Results.

In this section, we provide specific numerical examples to illustrate the bounds obtained in the previous sections. In particular, we focus on scalar Gaussian channel setting, such that: $G_{1}\leftarrow \eta_{1}$; $G_{2}\leftarrow \eta_{2}$; $K_{0}\leftarrow P_{0}$; $K_{01}^{\prime }\leftarrow P_{01}^{\prime }$; $K_{02}^{\prime }\leftarrow P_{02}^{\prime }$; $K_{1}\leftarrow P_{1}$; $K_{2}\leftarrow P_{2}$, $K_{S_{1}}\leftarrow Q_{1}$; $K_{S_{2}}\leftarrow Q_{2}$. We also denote $\left(A_{11},A_{12},A_{20},A_{21},A_{22},B_{1},B_{2}\right)\leftarrow \left(\alpha_{11},\alpha_{12},\alpha_{20},\alpha_{21},\alpha_{22},\beta_{1},\beta_{2}\right)$. We plot the inner and outer bounds for various values of helper power $P_{0}$, channel gains, $\eta_{1}$ and $\eta_{2}$ and different state power. The results are shown in Figure 12. The outer bound is based on Proposition 4. The inner bound is the convex hull of all the achievable regions, with interchange between the roles of the decoders. The time-sharing inner bound is according to point-to-point helper channel achievable region [28]. The scenario where the helper power is less than the users power is depicted in Figure 12a,b, while the channel gains in Figure 12a are equal, they are mismatched in Figure 12b. Please note that in both cases our inner bound outperforms the time-sharing bound, especially in the mismatched case, and some segments of the capacity region are characterized.

The scenario with helper power being higher than the user power and matched and mismatched channel gain is depicted in Figure 12c,d respectively. Similar to for low helper power regime, our proposed achievability scheme performs better than time-sharing.

## 5. Conclusions

In the first part of this paper, we have studied the parallel state-dependent Gaussian channel with a state-cognitive helper and with same but differently scaled states. An inner bound was derived and was compared to an upper bound, and the segments of the capacity region boundary were characterized for various channel parameters. We have shown that if the channel gain matrices satisfy a certain symmetry property, the full rectangular capacity region of the two point-to-point channels without the state can be achieved. Furthermore, for the scalar channel case, we have shown that for a given ratio of state gain over the helper signal gain, ab, one can find a value of the helper power—$P_{0}$, such that the capacity region is fully characterized.

A different model of the parallel state-dependent Gaussian channel with a state-cognitive helper and independent states was considered in the second part of this study. Inner and outer bounds were derived, and segments of the capacity region boundary were characterized for various channel parameters. We have also demonstrated our results using numerical simulation and have shown that our achievability scheme outperforms time-sharing that was shown to be optimal for the infinite state power regime in [34].

These two models represent a special case of a more general scenario with correlated states, our results in both studies imply that as the states get more correlated, it is easier to mitigate the interference. Furthermore, the gap between the inner bound and the outer bound in this work suggests that a new techniques for outer bound derivation is needed as we believe that the inner bounds consisting of pairs $\left(R_{1},R_{2}\right)=\left(f_{1}\left({\bar{A}}_{1}^{a},\bar{B},K_{01}^{\prime },K_{02}^{\prime }\right),f_{2}\left({\bar{A}}_{2},\bar{B},K_{01}^{\prime },K_{02}^{\prime }\right)\right)$ is indeed tight for some set of channel parameters.

## Appendix A. Proof of Proposition 1

Fix the following joint PMF

(A1) $$P_{SWX_{0}X_{1}X_{2}Y_{1}Y_{2}}=P_{S}P_{W|S}P_{X_{0}|SW}P_{X_{1}}P_{X_{2}}P_{Y_{1}|SX_{0}X_{1}}P_{Y_{2}|SX_{0}X_{2}}.$$

### Appendix A.1. Codebook Generation

Randomly and independently generate $2^{n\tilde{R}}$ sequences $w^{n}\left(\tilde{m}\right)$, $\tilde{m}\in I_{\tilde{R}}^{\left(n\right)}$, each according to $\prod_{i=1}^{n}P_{W}\left(w_{i}\right)$. Similarly, for l={1,2}, generate $2^{nR_{l}}$ sequences $x_{l}^{n}\left(m_{l}\right)$, $m_{l}\in I_{R_{l}}^{\left(n\right)}$, each according to $\prod_{i=1}^{n}P_{X_{l}}\left(x_{li}\right)$. These sequences constitute the codebook, which is revealed to the encoders and the decoders.

### Appendix A.2. Encoding

#### Appendix A.2.1. Encoder at the Helper

Fix $\epsilon^{\prime }>0$. Given $s^{n}$, find ${\tilde{m}}^{\prime }$, such that $\left(w^{n}\left({\tilde{m}}^{\prime }\right),s^{n}\right)\in T_{\epsilon^{\prime }}^{\left(n\right)}\left(P_{SW}\right)$. If no such sequence exists, it declares an error. Then, given $\left(w^{n}\left({\tilde{m}}^{\prime }\right),s^{n}\right)$, generate $x_{0}^{n}$ according to $\prod_{i=1}^{n}P_{X_{0}|SW}\left(x_{0i}|s_{i},w_{i}\left({\tilde{m}}^{\prime }\right)\right)$. The encoder at the helper then transmits $x_{0i}$ at time i∈[1:n].

#### Appendix A.2.2. Encoder at Transmitter l

To send message $m_{l}$, encoder *l* transmits $x_{l}^{n}\left(m_{l}\right)$, for l={1,2}.

### Appendix A.3. Decoding

Let $\epsilon >\epsilon^{\prime }$ and l∈{1,2}. Upon receiving $y_{l}^{n}$, the decoder at Receiver *l* declares that ${\hat{m}}_{l}\in I_{R_{l}}^{\left(n\right)}$ is sent if it is the unique message such that $\left(w^{n}\left(\hat{m}\right),x_{l}^{n}\left({\hat{m}}_{l}\right),y_{l}^{n}\right)\in T_{\epsilon }^{\left(n\right)}\left(P_{WX_{l}Y_{l}}\right)$ for some $\hat{m}\in I_{\tilde{R}}^{\left(n\right)}$; otherwise it declares an error.

### Appendix A.4. Analysis of the Probability of Error

The encoder at the helper declares an error if the following event occurs

(A2) $$E_{0}=\left\{\left(S^{n},W^{n}\left(\tilde{m}\right)\right)\notin T_{\epsilon^{\prime }}^{\left(n\right)}\left(P_{SW}\right)\;\mathrm{for}\;\mathrm{all}\;\tilde{m}\in I_{\tilde{R}}^{\left(n\right)}\right\}.$$

By the covering lemma (Section 2.3), with setting the original random variables $\left(U,X,\hat{X}\right)$ as (∅,S,W) respectively, and $A=I_{\tilde{R}}^{\left(n\right)}$, $P\{E_{0}\}$ tends to zero as n→∞ if

(A3) $$\tilde{R}>I\left(W;S\right).$$

Assume without loss of generality that $\left(M_{1},M_{2}\right)=\left(1,1\right)$, condition (A3) holds, and let $\tilde{M}$ denote the index of the chosen $w^{n}$ sequence for $s^{n}$. The decoder at Receiver *l* makes an error only if one or more of the following events occur:

$$\begin{array}{cc} E_{l1} & =\{\left(W^{n}\left(\tilde{M}\right),X_{l}^{n}\left(1\right),Y_{l}^{n}\right)\notin T_{\epsilon }^{\left(n\right)}\left(P_{WX_{l}Y_{l}}\right)\},\\ E_{l2} & =\{\left(W^{n}\left(\tilde{M}\right),X_{l}^{n}\left(m_{l}\right),Y_{l}^{n}\right)\in T_{\epsilon }^{\left(n\right)}\left(P_{WX_{l}Y_{l}}\right)\;\mathrm{for}\;\mathrm{some}\;m_{l}\neq l\},\\ E_{l3} & =\{\left(W^{n}\left(\tilde{m}\right),X_{l}^{n}\left(m_{l}\right),Y_{l}^{n}\right)\in T_{\epsilon }^{\left(n\right)}\left(P_{WX_{l}Y_{l}}\right)\;\mathrm{for}\;\mathrm{some}\;m_{l}\neq 1\;\mathrm{and}\;\tilde{m}\neq \tilde{M}\}. \end{array}$$

Thus, by the union of events bound,

(A4) $$P\left\{E_{l}\right\}=P\left\{E_{l1}\cup E_{l2}\cup E_{l3}\right\}\leq P\left\{E_{l1}\right\}+P\left\{E_{l2}\right\}+P\left\{E_{l3}\right\}.$$

By the LLN, the first term $P\{E_{l1}\}$ tend to zero as n→∞. For the second term, note that for $m_{l}\neq 1$,

$$\begin{array}{l} p(w^{n}\left(\tilde{M}\right),x_{l}^{n}\left(m_{l}\right),y_{l}^{n})\\ =\sum_{x_{l}^{n}\left(1\right),x_{0}^{n}\left(\tilde{M}\right),s^{n}}p\left(w^{n}\left(\tilde{M}\right),x_{0}^{n}\left(\tilde{M}\right),x_{l}^{n}\left(1\right),x_{l}^{n}\left(m_{l}\right),s^{n},y_{l}^{n}\right)\\ =p\left(x_{l}^{n}\left(m_{l}\right)\right)\sum_{x_{l}^{n}\left(1\right),x_{0}^{n}\left(\tilde{M}\right),s^{n}}p\left(w^{n}\left(\tilde{M}\right),x_{0}^{n}\left(\tilde{M}\right),s^{n}\right)p\left(x_{l}^{n}\left(1\right)\right)p\left(y_{l}^{n}|x_{0}^{n}\left(\tilde{M}\right),x_{l}^{n}\left(1\right),s^{n}\right)\\ =\prod_{i=1}^{n}p\left(x_{li}\left(m_{l}\right)\right)\sum_{x_{l}^{n}\left(1\right),x_{0}^{n}\left(\tilde{M}\right),s^{n}}\prod_{i=1}^{n}p\left(w_{i}\left(\tilde{M}\right),x_{0i}\left(\tilde{M}\right),s_{i}\right)p\left(x_{li}\left(1\right)\right)p\left(y_{li}|x_{0i}\left(\tilde{M}\right),x_{li}\left(1\right),s_{i}\right)\\ =\prod_{i=1}^{n}p\left(x_{li}\left(m_{l}\right)\right)p\left(w_{i}\left(\tilde{M}\right),y_{li}\right). \end{array}$$

Hence, by the packing lemma, choosing the original random variable (U,X,Y) as $\left(\emptyset ,X_{l},\left(W,Y_{l}\right)\right)$ respectively, $A=I_{R_{l}}^{\left(n\right)}$, $P\{E_{l2}\}$ tends to zero as n→∞ if $R_{l}<I\left(X_{l};Y_{l},W\right)$. Since $X_{l}$ and W are independent, $R_{l}<I\left(X_{l};Y_{l}|W\right)$. Finally, for the third term, note that for $m_{l}\neq 1$ and $\tilde{m}\neq \tilde{M}$

$$\begin{array}{l} p(w^{n}\left(\tilde{m}\right),x_{l}^{n}\left(m_{l}\right),y_{l}^{n})\\ =\sum_{w^{n}\left(\tilde{M}\right),x_{l}^{n}\left(1\right),x_{0}^{n}\left(\tilde{M}\right),s^{n}}p\left(w^{n}\left(\tilde{m}\right),w^{n}\left(\tilde{M}\right),x_{0}^{n}\left(\tilde{M}\right),x_{l}^{n}\left(1\right),x_{l}^{n}\left(m_{l}\right),s^{n},y_{l}^{n}\right)\\ =p\left(x_{l}^{n}\left(m_{l}\right)\right)p\left(w^{n}\left(\tilde{m}\right)\right)\sum_{w^{n}\left(\tilde{M}\right),x_{l}^{n}\left(1\right),x_{0}^{n}\left(\tilde{M}\right),s^{n}}p\left(w^{n}\left(\tilde{M}\right),x_{0}^{n}\left(\tilde{M}\right),s^{n}\right)p\left(x_{l}^{n}\left(1\right)\right)p\left(y_{l}^{n}|x_{0}^{n}\left(\tilde{M}\right),x_{l}^{n}\left(1\right),s^{n}\right)\\ =\prod_{i=1}^{n}p\left(x_{li}\left(m_{l}\right)\right)p\left(w_{i}\left(\tilde{m}\right)\right)p\left(y_{li}\right). \end{array}$$

Again, by the packing lemma, choosing the original random variable (U,X,Y) as $\left(\emptyset ,\left(W,X_{l}\right),Y_{l}\right)$ respectively, $A=I_{R_{l}}^{\left(n\right)}\times I_{\tilde{R}}^{\left(n\right)}$, $P\{E_{l3}\}$ tends to zero as n→∞ if $\tilde{R}+R_{l}<I\left(W,X_{l};Y_{l}\right)$.

## Appendix B. Proof of Proposition 3

We prove for a general l∈{1,2}. By Fano’s inequality (Lemma 2),

$$H\left(M_{l}|Y_{l}^{n}\right)\leq nR_{l}P_{e}^{\left(n\right)}+1\leq n\epsilon_{n}$$

where $\epsilon_{n}$ tends to zero as n→∞ by the assumption that $\lim_{n\to \infty }P_{e}^{\left(n\right)}=0$.

Now consider

$$\begin{array}{c} nR_{l}+I\left(S^{n};Y_{l}^{n}|M_{l}\right)\\ =H\left(M_{l}\right)-H\left(M_{l}|Y_{l}^{n}\right)+H\left(M_{l}|Y_{l}^{n}\right)+I\left(S^{n};Y_{l}^{n}|M_{l}\right)\\ \leq I\left(M_{l},S^{n};Y_{l}^{n}\right)+n\epsilon_{n}\\ =h\left(Y_{l}^{n}\right)-h\left(Y_{l}^{n}|M_{l},S^{n}\right)+n\epsilon_{n}\\ \leq h\left(Y_{l}^{n}\right)-h\left(Y_{l}^{n}|M_{l},X_{l}^{n},X_{0}^{n},S^{n}\right)+n\epsilon_{n}\\ =h\left(Y_{l}^{n}\right)-h\left(Z_{l}^{n}\right)+n\epsilon_{n}\\ \leq \frac{n}{2}\log\left(|G_{l}K_{0}G_{l}^{T}+K_{l}+G_{l}\Sigma_{X_{0}S}G_{s_{l}}^{T}+G_{s_{l}}\Sigma_{X_{0}S}^{T}G_{l}^{T}+G_{s_{l}}K_{S}G_{s_{l}}^{T}+I|\right)+n\epsilon_{n}. \end{array}$$

$I(S^{n};Y_{l}^{n}|M_{l})$ can be lower bounded as follows:

(A5) $$I\left(S^{n};Y_{l}^{n}|M_{l}\right)=h\left(S^{n}\right)-h\left(S^{n}|{\tilde{Y}}_{l}^{n}\right),$$

where ${\tilde{Y}}_{l}^{n}≜G_{l}X_{0}^{n}+G_{s_{l}}S^{n}+Z_{l}^{n}$. The conditional differential entropy can be upper bounded as follows

(A6) $$\begin{array}{cc} h(S^{n}|{\tilde{Y}}_{l}^{n}) & \leq \sum_{i=1}^{n}h\left(S_{i}|{\tilde{Y}}_{li}\right)\\ & \leq \frac{n}{2}\log{\left(2\pi e\right)}^{t}\left|K_{S}-\Sigma_{S{\tilde{Y}}_{l}}\Sigma_{{\tilde{Y}}_{l}}^{-1}\Sigma_{S{\tilde{Y}}_{l}}^{T}\right| \end{array}$$

where $\Sigma_{S{\tilde{Y}}_{l}}=E_{}\left[S{\tilde{Y}}_{l}^{T}\right]=K_{S}G_{s_{l}}^{T}+\Sigma_{X_{0}S}^{T}G_{l}^{T}$, and

$$\Sigma_{{\tilde{Y}}_{l}}=G_{l}K_{0}G_{l}^{T}+G_{l}\Sigma_{X_{0}S}G_{s_{l}}^{T}+G_{s_{l}}\Sigma_{X_{0}S}^{T}G_{l}^{T}+G_{s_{l}}K_{S}G_{s_{l}}^{T}+I.$$

Now we apply Silvester’s Determinant Theorem [38] to have

$$\begin{array}{cc} \left|K_{S}-\Sigma_{S{\tilde{Y}}_{l}}\Sigma_{{\tilde{Y}}_{l}}^{-1}\Sigma_{S{\tilde{Y}}_{l}}^{T}\right| & =\left|K_{S}\right|\left|I-K_{S}^{-1}\Sigma_{S{\tilde{Y}}_{l}}\Sigma_{{\tilde{Y}}_{l}}^{-1}\Sigma_{S{\tilde{Y}}_{l}}^{T}\right|\\ & =\left|K_{S}\right|\left|I-\Sigma_{S{\tilde{Y}}_{l}}^{T}K_{S}^{-1}\Sigma_{S{\tilde{Y}}_{l}}\Sigma_{{\tilde{Y}}_{l}}^{-1}\right|\\ & =\left|K_{S}\right|\left|\Sigma_{{\tilde{Y}}_{l}}-\Sigma_{S{\tilde{Y}}_{l}}^{T}K_{S}^{-1}\Sigma_{S{\tilde{Y}}_{l}}\right|\left|\Sigma_{{\tilde{Y}}_{l}}^{-1}\right|. \end{array}$$

Consider the argument of the middle determinant. Since $K_{S}^{-1}\Sigma_{S{\tilde{Y}}_{l}}=G_{s_{l}}^{T}+K_{S}^{-1}\Sigma_{X_{0}S}^{T}G_{l}^{T}$, it follows that

$$\begin{array}{cc} \Sigma_{S{\tilde{Y}}_{l}}^{T}K_{S}^{-1}\Sigma_{S{\tilde{Y}}_{l}} & =\left[G_{s_{l}}K_{S}+G_{l}\Sigma_{X_{0}S}\right]\left[G_{s_{l}}^{T}+K_{S}^{-1}\Sigma_{X_{0}S}^{T}G_{l}^{T}\right]\\ & =G_{S_{l}}K_{S}G_{S_{l}}^{T}+G_{S_{l}}\Sigma_{X_{0}S}^{T}G_{l}^{T}+G_{l}\Sigma_{X_{0}S}G_{S_{l}}^{T}+G_{l}\Sigma_{X_{0}S}K_{S}^{-1}\Sigma_{X_{0}S}^{T}G_{l}^{T}, \end{array}$$

and

$$\begin{array}{c} \Sigma_{{\tilde{Y}}_{l}}-\Sigma_{S{\tilde{Y}}_{l}}^{T}K_{S}^{-1}\Sigma_{S{\tilde{Y}}_{l}}=G_{l}\left(K_{0}-\Sigma_{X_{0}S}K_{S}^{-1}\Sigma_{X_{0}S}^{T}\right)G_{l}^{T}+I. \end{array}$$

Finally, by collecting terms,

$$\begin{array}{c} I\left(S^{n};Y_{l}^{n}|M_{l}\right)\geq \frac{n}{2}\log\left(\frac{|G_{l}K_{0}G_{l}^{T}+G_{l}\Sigma_{X_{0}S}G_{s_{l}}^{T}+G_{s_{l}}\Sigma_{X_{0}S}^{T}G_{l}^{T}+G_{s_{l}}K_{S}G_{s_{l}}^{T}+I|}{|G_{l}\left(K_{0}-\Sigma_{X_{0}S}K_{S}^{-1}\Sigma_{X_{0}S}^{T}\right)G_{l}^{T}+I|}\right). \end{array}$$

Thus, the bound in (11) is satisfied.

It remains to show that $\Sigma_{X_{0}S}K_{S}^{-1}\Sigma_{X_{0}S}^{T}⪯K_{0}$. We use the non-negativity property of the covariance matrix of the vector ${\left(X_{0},S\right)}^{T}$

$$\begin{array}{cc} \det E_{}\left[{\left(X_{0},S\right)}^{T}\left(X_{0},S\right)\right] & =\det\left[\begin{array}{cc} K_{0} & \Sigma_{X_{0}S}\\ \Sigma_{X_{0}S}^{T} & K_{S} \end{array}\right]\\ & =|K_{S}|\cdot \left|K_{0}-\Sigma_{X_{0}S}K_{S}^{-1}\Sigma_{X_{0}S}^{T}\right|\\ & \geq 0, \end{array}$$

where the last inequality follows since any covariance matrix ($\Sigma_{X_{0}S}$) is by definition positive definite. Now we arrange parts to have:

$$\begin{array}{c} \Sigma_{X_{0}S}K_{S}^{-1}\Sigma_{X_{0}S}^{T}⪯K_{0}. \end{array}$$

This completes the proof of Proposition 3.

## Appendix C. Optimal Coefficients for the MIMO Gaussian with Differently Scaled States Channel

We first consider the bound on $R_{1}$. Consider the first argument in min of (7a)

$$\begin{array}{cc} I\left(W,X_{1};Y_{1}\right)-I\left(W;S\right) & =I\left(X_{1};Y_{1}\right)+I\left(W;Y_{1}|X_{1}\right)-I\left(W;S|X_{1}\right)\\ & =I\left(X_{1};Y_{1}\right)+h\left(W|S,X_{1}\right)-h\left(W|X_{1},Y_{1}\right). \end{array}$$

It is straightforward to show that

$$\begin{array}{cc} I(X_{1};Y_{1}) & =\frac{1}{2}\log\left(\frac{|G_{1}K_{0}G_{1}^{T}+K_{1}+G_{1}BK_{S}G_{s_{1}}^{T}+G_{s_{1}}K_{S}B^{T}G_{1}^{T}+G_{s_{1}}K_{S}G_{s_{1}}^{T}+I|}{|G_{1}K_{0}G_{1}^{T}+G_{1}BK_{S}G_{s_{1}}^{T}+G_{s_{1}}K_{S}B^{T}G_{1}^{T}+G_{s_{1}}K_{S}G_{s_{1}}^{T}+I|}\right), \end{array}$$

and

$$\begin{array}{cc} h(W|S,X_{1}) & =h(X_{0}^{\prime }). \end{array}$$

As for the third term, denote ${\tilde{Y}}_{1}=Y_{1}-X_{1}$, thus

$$\begin{array}{cc} h(W|X_{1},Y_{1}) & =h(W|{\tilde{Y}}_{1})\\ & =h(W-M_{W|{\tilde{Y}}_{1}}{\tilde{Y}}_{1})\\ & =h\left(X_{0}^{\prime }+AS-M_{W|{\tilde{Y}}_{1}}\left(G_{1}\left(X_{0}^{\prime }+BS\right)+G_{s_{1}}S+Z_{1}\right)\right). \end{array}$$

We require that term S in the argument of the differential entropy be completely canceled, therefore we choose

$$\begin{array}{cc} A^{a} & =M_{W|{\tilde{Y}}_{1}}\left(G_{1}B+G_{s_{1}}\right). \end{array}$$

With the above choice of *A*, we have

$$h\left(W|X_{1},Y_{1}\right)=h\left(X_{0}^{\prime }-M_{W|{\tilde{Y}}_{1}}\left(G_{1}X_{0}^{\prime }+Z_{1}\right)\right).$$

Finally, we demand that $M_{W|{\tilde{Y}}_{1}}$ be the MMSE of $X_{0}^{\prime }$ given $G_{1}X_{0}^{\prime }+Z_{1}$, i.e.,

$$M_{W|{\tilde{Y}}_{1}}={\left(G_{1}K_{0}^{\prime }G_{1}^{T}+I\right)}^{-1}K_{0}^{\prime }G_{1}^{T}.$$

In such case

$$\begin{array}{cc} A^{a} & ={\left(G_{1}K_{0}^{\prime }G_{1}^{T}+I\right)}^{-1}K_{0}^{\prime }G_{1}^{T}\left(G_{1}B+G_{s_{1}}\right). \end{array}$$

Hence

$$h\left(W|X_{1},Y_{1}\right)=h\left(X_{0}^{\prime }|G_{1}X_{0}^{\prime }+Z_{1}\right),$$

and thus

$$\begin{array}{cc} h\left(W|S,X_{1}\right)-h\left(W|X_{1},Y_{1}\right) & =h\left(X_{0}^{\prime }\right)-h\left(X_{0}^{\prime }|G_{1}X_{0}^{\prime }+Z_{1}\right)\\ & =I(X_{0}^{\prime };G_{1}X_{0}^{\prime }+Z_{1})\\ & =\frac{1}{2}\log\left(|G_{1}K_{0}^{\prime }G_{1}^{T}+I|\right). \end{array}$$

Furthermore, if we choose $A^{b}=B+G_{1}^{-1}G_{s_{1}}$, then

$$\begin{array}{cc} I(X_{1};Y_{1}|W) & =I(X_{1};G_{1}\left(X_{0}^{\prime }+BS\right)+G_{s_{1}}S+X_{1}+Z_{1}|X_{0}^{\prime }+\left(B+G_{1}^{-1}G_{s_{1}}\right)S)\\ & =I(X_{1};X_{1}+Z_{1})\\ & =\frac{1}{2}\log\left(|K_{1}+I|\right). \end{array}$$

With this choice of *A*, $h(W|{\tilde{Y}}_{1})$ is equal to

$$\begin{array}{c} h(W|{\tilde{Y}}_{1})\\ =h(X_{0}^{\prime }+AS|G_{1}\left(X_{0}^{\prime }+\left(A-G_{1}^{-1}G_{s_{1}}\right)S\right)+G_{s_{1}}S+Z_{1})\\ =h(X_{0}^{\prime }+AS|G_{1}\left(X_{0}^{\prime }+AS\right)+Z_{1})\\ =\frac{1}{2}\log{\left(2\pi e\right)}^{t}\;\left|K_{0}^{\prime }\;+\;AK_{S}A^{T}\;-\;\left(K_{0}^{\prime }G_{1}^{T}\;+\;AK_{S}A^{T}G_{1}^{T}\right){\left(G_{1}\left(K_{0}^{\prime }\;+\;AK_{S}A^{T}\right)G_{1}^{T}\;+\;I\right)}^{-1}{\left(K_{0}^{\prime }G_{1}^{T}\;+\;AK_{S}A^{T}G_{1}^{T}\right)}^{T}\right|\\ =\frac{1}{2}\log{\left(2\pi e\right)}^{t}\;\left|K_{0}^{\prime }+AK_{S}A^{T}-\left(K_{0}^{\prime }+AK_{S}A^{T}\right)G_{1}^{T}{\left(G_{1}\left(K_{0}^{\prime }+AK_{S}A^{T}\right)G_{1}^{T}+I\right)}^{-1}G_{1}{\left(K_{0}^{\prime }+AK_{S}A^{T}\right)}^{T}\right|\\ =\frac{1}{2}\log{\left(2\pi e\right)}^{t}\;\left|K_{0}^{\prime }+AK_{S}A^{T}\right|\left|I-G_{1}^{T}{\left(G_{1}\left(K_{0}^{\prime }+AK_{S}A^{T}\right)G_{1}^{T}+I\right)}^{-1}G_{1}{\left(K_{0}^{\prime }+AK_{S}A^{T}\right)}^{T}\right|\\ =\frac{1}{2}\log{\left(2\pi e\right)}^{t}\;\left|K_{0}^{\prime }+AK_{S}A^{T}\right|\left|I-{\left(G_{1}\left(K_{0}^{\prime }+AK_{S}A^{T}\right)G_{1}^{T}+I\right)}^{-1}G_{1}{\left(K_{0}^{\prime }+AK_{S}A^{T}\right)}^{T}G_{1}^{T}\right|\\ =\frac{1}{2}\log{\left(2\pi e\right)}^{t}\frac{\left|K_{0}^{\prime }+AK_{S}A^{T}\right|}{\left|G_{1}\left(K_{0}^{\prime }+AK_{S}A^{T}\right)G_{1}^{T}+I\right|}\left|\left(G_{1}\left(K_{0}^{\prime }+AK_{S}A^{T}\right)G_{1}^{T}+I\right)-G_{1}{\left(K_{0}^{\prime }+AK_{S}A^{T}\right)}^{T}G_{1}^{T}\right|\\ =\frac{1}{2}\log{\left(2\pi e\right)}^{t}\frac{\left|K_{0}^{\prime }+AK_{S}A^{T}\right|}{\left|G_{1}\left(K_{0}^{\prime }+AK_{S}A^{T}\right)G_{1}^{T}+I\right|}. \end{array}$$

Thus

$$\begin{array}{cc} I\left(W,X_{1};Y_{1}\right)-I\left(W;S\right) & =\frac{1}{2}\log\left(\frac{|K_{0}^{\prime }\left|\right|G_{1}\left(K_{0}^{\prime }+AK_{S}A^{T}\right)G_{1}^{T}+K_{1}+I|}{|K_{0}^{\prime }+AK_{S}A^{T}|}\right). \end{array}$$

We would like to obtain a condition under which $g_{1}\left(A^{b},B,K_{0}^{\prime }\right)\leq f_{1}\left(A^{b},B,K_{0}^{\prime }\right)$, i.e.,

$$\begin{array}{c} |K_{0}^{\prime }\left|\right|G_{1}\left(K_{0}^{\prime }+AK_{S}A^{T}\right)G_{1}^{T}+K_{1}+I|\geq |K_{0}^{\prime }+AK_{S}A^{T}\left|\right|K_{1}+I| \end{array}$$

that is equivalent to

$$\begin{array}{c} K_{0}^{\prime }G_{1}\left(K_{0}^{\prime }+AK_{S}A^{T}\right)G_{1}^{T}+K_{0}^{\prime }K_{1}+K_{0}^{\prime }⪰K_{0}^{\prime }K_{1}+K_{0}^{\prime }+AK_{S}A^{T}K_{1}+AK_{S}A^{T}. \end{array}$$

Furthermore, after rearranging terms, we have

$$\begin{array}{c} K_{0}^{\prime }G_{1}K_{0}^{\prime }G_{1}^{T}⪰AK_{S}A^{T}\left(K_{1}+I\right)-K_{0}^{\prime }G_{1}AK_{S}A^{T}G_{1}^{T}. \end{array}$$

The choices of $A^{c}$ and $A^{d}$ for the achievability proof of $R_{2}$ follows using similar steps by interchanging the indices 1←2.

## Appendix D. Proof of Proposition 4

This proof relies greatly on the proof of the outer bound in the differently scaled states scenario in Appendix B. The main differences are:

- $S={\left(S_{1},S_{2}\right)}^{T}$. Since $S_{1}$ and $S_{2}$ are independent, the covariance matrix of S is block-diagonal
  $$K_{S}=\left(\begin{array}{cc} K_{S_{1}} & 0\\ 0 & K_{S_{2}} \end{array}\right),$$
- the helper signal $X_{0}$ correlates with $S_{1}$ and $S_{2}$ and it characterized by the cross-covariance matrices $\Sigma_{X_{0}S_{1}}$ and $\Sigma_{X_{0}S_{2}}$ respectively,
- the state gain matrices, $G_{s_{1}}$ and $G_{s_{2}}$ are unity matrices.

Hence, in the independent-states case, we have the following upper bound on $nR_{1}+I\left(S^{n};Y_{1}^{n}|M_{1}\right)$

(A7) $$nR_{1}+I\left(S^{n};Y_{1}^{n}|M_{1}\right)\leq \frac{n}{2}\log\left(|G_{1}K_{0}G_{1}^{T}+K_{1}+G_{1}\Sigma_{X_{0}S_{1}}+\Sigma_{X_{0}S_{1}}^{T}G_{1}^{T}+K_{S_{1}}+I|\right)+n\epsilon_{n}.$$

Let ${\tilde{Y}}_{1}^{n}≜G_{1}X_{0}^{n}+S_{1}^{n}+Z_{1}^{n}$. We proceed to lower bound $I\left(S^{n};Y_{1}^{n}|M_{1}\right)=h\left(S^{n}\right)-h\left(S^{n}|{\tilde{Y}}_{1}^{n}\right)$ In similar fashion to (A6), the conditional differential entropy can be upper bounded as follows

(A8) $$h\left(S^{n}|{\tilde{Y}}_{1}^{n}\right)\leq \frac{n}{2}\log{\left(2\pi e\right)}^{2t}\left|K_{S}\right|\left|\Sigma_{{\tilde{Y}}_{l}}-K_{S{\tilde{Y}}_{l}}^{T}\Sigma_{S}^{-1}\Sigma_{S{\tilde{Y}}_{l}}\right|\left|\Sigma_{{\tilde{Y}}_{1}}^{-1}\right|,$$

where

$$\begin{array}{cc} \Sigma_{S{\tilde{Y}}_{1}} & =E_{}\left[S{\tilde{Y}}_{1}\right]=\left[\begin{array}{c} K_{S_{1}}+\Sigma_{X_{0}S_{1}}^{T}G_{1}^{T}\\ \Sigma_{X_{0}S_{2}}^{T}G_{1}^{T} \end{array}\right], \end{array}$$

and $\Sigma_{{\tilde{Y}}_{1}}=G_{1}K_{0}G_{1}^{T}+G_{1}\Sigma_{X_{0}S_{1}}+\Sigma_{X_{0}S_{1}}^{T}G_{1}^{T}+K_{S_{1}}+I$. The power 2t in (A8) is due to the size of the vector ${\left(S_{1},S_{2}\right)}^{T}$. The argument of the inner determinant in (A8) can be further evaluated as follows,

$$\begin{array}{cc} \Sigma_{S}^{-1}\Sigma_{S{\tilde{Y}}_{1}} & =\left[\begin{array}{cc} K_{S_{1}}^{-1} & 0\\ 0 & K_{S_{2}}^{-1} \end{array}\right]\left[\begin{array}{c} K_{S_{1}}+\Sigma_{X_{0}S_{1}}^{T}G_{1}^{T}\\ \Sigma_{X_{0}S_{2}}^{T}G_{1}^{T} \end{array}\right]\\ & =\left[\begin{array}{c} I+K_{S_{1}}^{-1}\Sigma_{X_{0}S_{1}}^{T}G_{1}^{T}\\ K_{S_{2}}^{-1}\Sigma_{X_{0}S_{2}}^{T}G_{1}^{T} \end{array}\right], \end{array}$$

and

$$\begin{array}{cc} \Sigma_{S{\tilde{Y}}_{1}}^{T}\Sigma_{S}^{-1}\Sigma_{S{\tilde{Y}}_{1}} & =\left[\begin{array}{cc} K_{S_{1}}+G_{1}\Sigma_{X_{0}S_{1}} & G_{1}\Sigma_{X_{0}S_{2}} \end{array}\right]\left[\begin{array}{c} I+K_{S_{1}}^{-1}\Sigma_{X_{0}S_{1}}^{T}G_{1}^{T}\\ K_{S_{2}}^{-1}\Sigma_{X_{0}S_{2}}^{T}G_{1}^{T} \end{array}\right]\\ & =K_{S_{1}}+G_{1}\Sigma_{X_{0}S_{1}}+\Sigma_{X_{0}S_{1}}^{T}G_{1}^{T}+G_{1}\Sigma_{X_{0}S_{1}}K_{S_{1}}^{-1}\Sigma_{X_{0}S_{1}}^{T}G_{1}^{T}+G_{1}\Sigma_{X_{0}S_{2}}K_{S_{2}}^{-1}\Sigma_{X_{0}S_{2}}^{T}G_{1}^{T}. \end{array}$$

Thus,

$$\begin{array}{cc} \Sigma_{{\tilde{Y}}_{1}}-\Sigma_{S{\tilde{Y}}_{1}}^{T}\Sigma_{S}^{-1}\Sigma_{S{\tilde{Y}}_{1}} & =G_{1}\left(K_{0}-\Sigma_{X_{0}S_{1}}K_{S_{1}}^{-1}\Sigma_{X_{0}S_{1}}^{T}-\Sigma_{X_{0}S_{2}}K_{S_{2}}^{-1}\Sigma_{X_{0}S_{2}}^{T}\right)G_{1}^{T}+I\\ & =G_{1}\left(K_{0}-\Sigma_{X_{0}S}K_{S}^{-1}\Sigma_{X_{0}S}^{T}\right)G_{1}^{T}+I, \end{array}$$

where in the last equality we used the definition of $\Sigma_{X_{0}S}$ from (26). Consequently, we established an upper bound on $I(S^{n};Y_{1}^{n}|M_{1})$:

(A9) $$I\left(S^{n};Y_{1}^{n}|M_{1}\right)\geq \frac{n}{2}\log\left(\frac{|G_{1}K_{0}G_{1}^{T}+G_{1}\Sigma_{X_{0}S_{1}}+\Sigma_{X_{0}S_{1}}^{T}G_{1}^{T}+K_{S_{1}}+I|}{|G_{1}\left(K_{0}-\Sigma_{X_{0}S}K_{S}^{-1}\Sigma_{X_{0}S}^{T}\right)G_{1}^{T}+I|}\right).$$

Finally, collecting (A7) and (A9), the bound in (25), with l=1, is satisfied. The bound (25) for l=2 follows from similar considerations. It remains to show that $\Sigma_{X_{0}S}K_{S}^{-1}\Sigma_{X_{0}S}^{T}⪯K_{0}$. We use the non-negativity property of the covariance matrix of the vector ${\left(X_{0},S_{1},S_{2}\right)}^{T}$

$$\begin{array}{cc} \det E_{}\left[{\left(X_{0},S_{1},S_{2}\right)}^{T}\left(X_{0},S_{1},S_{2}\right)\right] & =\det\left[\begin{array}{ccc} K_{0} & \Sigma_{X_{0}S_{1}} & \Sigma_{X_{0}S_{2}}\\ \Sigma_{X_{0}S_{1}}^{T} & K_{S_{1}} & 0\\ \Sigma_{X_{0}S_{2}}^{T} & 0 & K_{S_{2}} \end{array}\right]\\ & =\left|\left[\begin{array}{cc} K_{S_{1}} & 0\\ 0 & K_{S_{2}} \end{array}\right]\right|\cdot \left|K_{0}-\left[\begin{array}{cc} \Sigma_{X_{0}S_{1}} & \Sigma_{X_{0}S_{2}} \end{array}\right]{\left[\begin{array}{cc} K_{S_{1}} & 0\\ 0 & K_{S_{2}} \end{array}\right]}^{-1}\left[\begin{array}{c} \Sigma_{X_{0}S_{1}}^{T}\\ \Sigma_{X_{0}S_{2}}^{T} \end{array}\right]\right|\\ & =\left|\left[\begin{array}{cc} K_{S_{1}} & 0\\ 0 & K_{S_{2}} \end{array}\right]\right|\cdot \left|K_{0}-\left[\begin{array}{cc} \Sigma_{X_{0}S_{1}} & \Sigma_{X_{0}S_{2}} \end{array}\right]\left[\begin{array}{cc} K_{S_{1}}^{-1} & 0\\ 0 & K_{S_{2}}^{-1} \end{array}\right]\left[\begin{array}{c} \Sigma_{X_{0}S_{1}}^{T}\\ \Sigma_{X_{0}S_{2}}^{T} \end{array}\right]\right|\\ & =\left|\left[\begin{array}{cc} K_{S_{1}} & 0\\ 0 & K_{S_{2}} \end{array}\right]\right|\cdot \left|K_{0}-\Sigma_{X_{0}S_{1}}K_{S_{1}}^{-1}\Sigma_{X_{0}S_{1}}^{T}-\Sigma_{X_{0}S_{2}}K_{S_{2}}^{-1}\Sigma_{X_{0}S_{2}}^{T}\right|\\ & \overset{\left(b\right)}{\geq }0, \end{array}$$

where the last inequality follows from non-negativity of covariance matrix. Now we arrange parts to have:

$$\Sigma_{X_{0}S_{1}}K_{S_{1}}^{-1}\Sigma_{X_{0}S_{1}}^{T}+\Sigma_{X_{0}S_{2}}K_{S_{2}}^{-1}\Sigma_{X_{0}S_{2}}^{T}=\Sigma_{X_{0}S}K_{S}^{-1}\Sigma_{X_{0}S}^{T}⪯K_{0}.$$

This completes the proof of Proposition 4.

## Appendix E. Proof of Proposition 5

We use random codes and fix the following joint distribution:

$$\begin{array}{c} P_{SUVX_{0}X_{1}X_{2}Y_{1}Y_{2}}=P_{SUV}P_{X_{0}|SUV}P_{X_{1}}P_{X_{2}}P_{Y_{1}|SX_{0}X_{1}}P_{Y_{2}|SX_{0}X_{2}}. \end{array}$$

### Appendix E.1. Codebook Generation

Generate $2^{n{\tilde{R}}_{U}}$ randomly and independently generated sequences $u^{n}\left(r\right)$, $r\in I_{{\tilde{R}}_{U}}^{\left(n\right)}$, each according to $\prod_{i=1}^{n}P_{U}\left(u_{i}\right)$. Similarly, generate $2^{n{\tilde{R}}_{V}}$ randomly and independently generated sequences $v^{n}\left(t\right)$, $t\in I_{{\tilde{R}}_{V}}^{\left(n\right)}$ according to $\prod_{i=1}^{n}P_{V}\left(v_{i}\right)$.

Let l∈{1,2}. Randomly and independently generate $2^{nR_{l}}$ sequences $x_{1}^{n}\left(m_{l}\right)$, $m_{l}\in I_{R_{l}}^{\left(n\right)}$, each according to $\prod_{i=1}^{n}P_{X_{l}}\left(x_{li}\right)$.

These sequences constitute the codebook, which is revealed to the encoders and the decoders.

### Appendix E.2. Encoding

Fix $\epsilon^{\prime \prime }>\epsilon^{\prime }>0$. The encoder at the helper, given $s^{n}$, finds $\tilde{r}$ such that

$$\left(s^{n},u^{n}\left(\tilde{r}\right)\right)\in T_{\epsilon^{\prime }}^{\left(n\right)}\left(P_{SU}\right),$$

if there is more than one such $\tilde{r}$, choose the smallest one. If no such $\tilde{r}$ can be found declare an error. Next, given $s^{n},u^{n}\left(\tilde{r}\right)$, find $\tilde{t}$ such that

$$\left(s^{n},u^{n}\left(\tilde{r}\right),v^{n}\left(\tilde{t}\right)\right)\in T_{\epsilon^{\prime \prime }}^{\left(n\right)}\left(P_{SUV}\right),$$

if there is more than one such $\tilde{t}$, choose the smallest one. If no such $\tilde{t}$ can be found declare an error. Then, given $s^{n}$, $u^{n}\left(\tilde{r}\right)$ and $v^{n}\left(\tilde{t}\right)$, generate $x_{0}^{n}$ with i.i.d. components according to $\prod_{i=1}^{n}P_{X_{0}|SUV}\left(x_{0i}|s_{i},u_{i},v_{i}\right)$. Let $\left(m_{1},m_{2}\right)$ be the messages to be sent. The encoder at transmitter *l* transmits $x_{l}^{n}\left(m_{l}\right)$.

### Appendix E.3. Decoding

Fix $\epsilon >\epsilon^{\prime \prime }$. Given $y_{1}^{n}$, decoder 1 declares that ${\hat{m}}_{1}$ was sent if it is the unique message such that

$$\left(u^{n}\left(\hat{r}\right),x_{1}^{n}\left({\hat{m}}_{1}\right),y_{1}^{n}\right)\in T_{\epsilon }^{\left(n\right)}\left(P_{UX_{1}Y_{1}}\right).$$

If no or more than one such ${\hat{m}}_{1}$ can be found, it declares an error.

Similarly, given $y_{2}^{n}$, decoder 2 finds the unique message ${\hat{m}}_{2}$ such that

$$\left(v^{n}\left(\hat{t}\right),x_{2}^{n}\left({\hat{m}}_{2}\right),y_{2}^{n}\right)\in T_{\epsilon }^{\left(n\right)}\left(P_{VX_{2}Y_{2}}\right).$$

If no or more than one such ${\hat{m}}_{2}$ can be found, it declares an error.

### Appendix E.4. Analysis of the Probability of Error

Assume without loss of generality that the message pair $\left(M_{1},M_{2}\right)=\left(1,1\right)$ was sent and let $r_{0}$ be the chosen index for $u^{n}$ and $t_{0}$ be the chosen index for $v^{n}$. The encoder at the helper makes an error only if one or both of the following errors occur:

$$\begin{array}{cc} E_{01} & =\{\left(S^{n},U^{n}\left(r\right)\right)\notin T_{\epsilon^{\prime }}^{\left(n\right)}\left(P_{SU}\right)\;\mathrm{for}\;\mathrm{all}\;r\in I_{{\tilde{R}}_{U}}^{\left(n\right)}\},\\ E_{02} & =\{\left(S^{n},U^{n}\left(r_{0}\right),V^{n}\left(t\right)\right)\notin T_{\epsilon^{\prime \prime }}^{\left(n\right)}\left(P_{SUV}\right)\;\mathrm{for}\;\mathrm{all}\;t\in I_{{\tilde{R}}_{V}}^{\left(n\right)}\}. \end{array}$$

Thus, by the union of events bound, the probability that the encoder at the helper makes an error, can be upper bounded as

$$\mathrm{Pr}\left(E_{0}\right)=\mathrm{Pr}\left(E_{01}\cup E_{02}\right)\leq \mathrm{Pr}\left(E_{01}\right)+\mathrm{Pr}\left(E_{01}^{c}\cap E_{02}\right).$$

By the covering lemma with U=∅, X←S, $\hat{X}\leftarrow U$, and $A=I_{{\tilde{R}}_{U}}^{\left(n\right)}$, $\mathrm{Pr}(E_{01})$ tends to zero as n→∞ if ${\tilde{R}}_{U}>I\left(U;S\right)+\delta \left(\epsilon^{\prime }\right)$.

Similarly, using the covering lemma with U=∅, X←(S,U), $\hat{X}\leftarrow V$, and $A=I_{{\tilde{R}}_{V}}^{\left(n\right)}$, $\mathrm{Pr}(E_{01}^{c}\cap E_{02})$ tends to zero as n→∞ if ${\tilde{R}}_{V}>I\left(V;S,U\right)+\delta \left(\epsilon^{\prime \prime }\right)$.

The decoder at receiver 1 makes an error only if one or more of the following events occur

$$\begin{array}{cc} E_{11} & =\{\left(U^{n}\left(r_{0}\right),X_{1}^{n}\left(1\right),Y_{1}^{n}\right)\notin T_{\epsilon }^{\left(n\right)}\left(P_{UX_{1}Y_{1}}\right)\},\\ E_{12} & =\{\left(U^{n}\left(r_{0}\right),X_{1}^{n}\left(m_{1}\right),Y_{1}^{n}\right)\in T_{\epsilon }^{\left(n\right)}\left(P_{UX_{1}Y_{1}}\right)\;\mathrm{for}\;\mathrm{some}\;m_{1}\neq 1\},\\ E_{13} & =\{\left(U^{n}\left(r\right),X_{1}^{n}\left(m_{1}\right),Y_{1}^{n}\right)\in T_{\epsilon }^{\left(n\right)}\left(P_{UX_{1}Y_{1}}\right)\;\mathrm{for}\;\mathrm{some}\;r\neq r_{0}\;\mathrm{and}\;m_{1}\neq 1\}. \end{array}$$

Again, by the union of events bound, the probability that the decoder at receiver 1 makes an error, can be upper bounded as

(A10) $$\begin{array}{cc} \mathrm{Pr}(E_{1}) & =\mathrm{Pr}(E_{11}\cup E_{12}\cup E_{13})\\ & \leq \mathrm{Pr}(E_{01}\cup E_{11}\cup E_{12}\cup E_{13})\\ & \leq \mathrm{Pr}\left(E_{01}\right)+\mathrm{Pr}\left(E_{01}^{c}\cap E_{11}\right)+\mathrm{Pr}\left(E_{01}^{c}\cap E_{12}\right)+\mathrm{Pr}\left(E_{13}\right). \end{array}$$

We have already shown that $\mathrm{Pr}(E_{01})$ tends to zero as n→∞ if ${\tilde{R}}_{U}>I\left(U;S\right)+\delta \left(\epsilon^{\prime }\right)$. Next, note that

$$E_{01}^{c}=\left\{\left(S^{n},U^{n}\left(r_{0}\right)\right)\in T_{\epsilon^{\prime }}^{\left(n\right)}\left(P_{SU}\right)\right\}=\left\{\left(S^{n},U^{n}\left(r_{0}\right),X_{0}^{n}\right)\in T_{\epsilon^{\prime }}^{\left(n\right)}\left(P_{SUX_{0}}\right)\right\},$$

and

$$\begin{array}{cc} P_{Y_{1}^{n}|S^{n}U^{n}\left(r_{0}\right)X_{0}^{n}X_{1}^{n}\left(1\right)}\left(y_{1}^{n}|s^{n},u^{n},x_{0}^{n},x_{1}^{n}\right) & =\prod_{i=1}^{n}P_{Y_{1}|SUX_{0}X_{1}}\left(y_{1i}|s_{i},u_{i},x_{0i},x_{1i}\right)\\ & =\prod_{i=1}^{n}P_{Y_{1}|SX_{0}X_{1}}\left(y_{1i}|s_{i},x_{0i},x_{1i}\right). \end{array}$$

Hence, by the conditionally typicality lemma, $\mathrm{Pr}(E_{01}^{c}\cap E_{11})$ tends to zero as n→∞.

As for the probability of the event $\left(E_{01}^{c}\cap E_{12}\right)$, $X_{1}^{n}\left(m_{1}\right)$ is independent of $\left(U^{n}\left(r_{0}\right),Y_{1}^{n}\right)∼\prod_{i=1}^{n}P_{UY_{1}}\left(u_{i},y_{1i}\right)$. Hence, by the packing lemma, with U=∅, $X\leftarrow X_{1}$, $Y\leftarrow (U,Y_{1})$ and $A=[2:2^{nR_{1}}]$, $\mathrm{Pr}(E_{01}^{c}\cap E_{12})$ tends to zero as n→ if $R_{1}<I\left(X_{1};U,Y_{1}\right)-\delta \left(\epsilon \right)$. $X_{1}$ and U are mutually independent, hence the latter condition is equivalent to $R_{1}<I\left(X_{1};Y_{1}|U\right)-\delta \left(\epsilon \right)$.

Finally, since for $m_{1}\neq 1$, $r\neq r_{0}$, $\left(X_{1}^{n}\left(m_{1}\right),U^{n}\left(r\right)\right)$ is independent of $\left(X_{1}^{n}\left(1\right),U^{n}\left(r_{0}\right),Y_{1}^{n}\right)$, again by the packing lemma with U=∅, $X\leftarrow (U,X_{1})$, $Y\leftarrow Y_{1}$ and $A=\left[2:2^{nR_{1}}\right]\times \left[1:2^{n{\tilde{R}}_{U}}\right]/r_{0}$, $\mathrm{Pr}(E_{13})$ tends to zero as n→∞ if ${\tilde{R}}_{U}+R_{1}<I\left(U,X_{1};Y_{1}\right)-\delta \left(\epsilon \right)$.

Next consider the average probability of error for decoder 2. The decoder at receiver 2 makes an error only if one or more of the following events occur

$$\begin{array}{cc} E_{21} & =\{\left(V^{n}\left(t_{0}\right),X_{2}^{n}\left(1\right),Y_{2}^{n}\right)\notin T_{\epsilon }^{\left(n\right)}\left(P_{VX_{2}Y_{2}}\right)\},\\ E_{22} & =\{\left(V^{n}\left(t_{0}\right),X_{2}^{n}\left(m_{2}\right),Y_{2}^{n}\right)\in T_{\epsilon }^{\left(n\right)}\left(P_{VX_{2}Y_{2}}\right)\;\mathrm{for}\;\mathrm{some}\;m_{2}\neq 1\},\\ E_{23} & =\{\left(V^{n}\left(t\right),X_{2}^{n}\left(m_{2}\right),Y_{2}^{n}\right)\in T_{\epsilon }^{\left(n\right)}\left(P_{VX_{2}Y_{2}}\right)\;\mathrm{for}\;\mathrm{some}\;t\neq t_{0}\;\mathrm{and}\;m_{2}\neq 1\}. \end{array}$$

Similar to (A10), the probability that the decoder at receiver 2 makes an error, can be upper bounded as

$$\mathrm{Pr}\left(E_{2}\right)\leq \mathrm{Pr}\left(E_{0}\right)+\mathrm{Pr}\left(E_{0}^{c}\cap E_{21}\right)+\mathrm{Pr}\left(E_{0}^{c}\cap E_{22}\right)+\mathrm{Pr}\left(E_{23}\right).$$

In a very similar fashion as was shown for decoder 1, it can be shown that $\mathrm{Pr}(E_{2})$ tends to zero as n→∞ if

$$\begin{array}{cc} {\tilde{R}}_{V}\geq & \;I\left(V;S,U\right)+\delta \left(\epsilon^{\prime \prime }\right),\\ R_{2}\leq & \;I\left(X_{2};Y_{2}|V\right)-\delta \left(\epsilon \right),\\ R_{2}+{\tilde{R}}_{V}\leq & \;I\left(V,X_{2};Y_{2}\right)-\delta \left(\epsilon \right). \end{array}$$

Finally, combining the aforementioned bounds yields the following achievable region:

$$\begin{array}{cc} R_{1}\leq & \;\min\left\{I\left(U,X_{1};Y_{1}\right)-I\left(U;S\right),I\left(X_{1};Y_{1}|U\right)\right\},\\ R_{2}\leq & \;\min\left\{I\left(V,X_{2};Y_{2}\right)-I\left(V;U,S\right),I\left(X_{2};Y_{2}|V\right)\right\}. \end{array}$$

This completes the proof of achievability.

## Appendix F. Optimal Coefficients for the MIMO Gaussian with Independent States Channel

We first consider the bound on $R_{1}$. Consider the first argument in min of (28a)

(A11) $$\begin{array}{cc} I\left(U,X_{1};Y_{1}\right)-I\left(U;S\right) & =I\left(X_{1};Y_{1}\right)+I\left(U;Y_{1}|X_{1}\right)-I\left(U;S|X_{1}\right)\\ & =I\left(X_{1};Y_{1}\right)+h\left(U|S,X_{1}\right)-h\left(U|X_{1},Y_{1}\right). \end{array}$$

It is straightforward to show that

$$\begin{array}{cc} I(X_{1};Y_{1}) & =\frac{1}{2}\log\left(\frac{|G_{1}K_{0}G_{1}^{T}+K_{1}+G_{1}B_{1}K_{S_{1}}+K_{S_{1}}B_{1}^{T}G_{1}^{T}+K_{S_{1}}+I|}{|G_{1}K_{0}G_{1}^{T}+G_{1}B_{1}K_{S_{1}}+K_{S_{1}}B_{1}^{T}G_{1}^{T}+I|}\right), \end{array}$$

and

$$h\left(U|S,X_{1}\right)=h\left(X_{01}^{\prime }\right)=\frac{1}{2}\log{\left(2\pi e\right)}^{t}\left|K_{01}^{\prime }\right|.$$

As for the third term in (A11), denote ${\tilde{Y}}_{1}=Y_{1}-X_{1}$,

$$\begin{array}{cc} h(U|X_{1},Y_{1}) & =h(U|{\tilde{Y}}_{1})\\ & =h(U-M_{U|{\tilde{Y}}_{1}}{\tilde{Y}}_{1})\\ & =h\left(X_{01}^{\prime }+A_{11}S_{1}+A_{12}S_{2}-M_{U|{\tilde{Y}}_{1}}\left(G_{1}\left(X_{01}^{\prime }+X_{02}^{\prime }+B_{1}S_{1}+B_{2}S_{2}\right)+S_{1}+Z_{1}\right)\right). \end{array}$$

We require that the terms $S_{1}$ and $S_{2}$ in the argument of the differential entropy be completely canceled, hence we choose

$$\begin{array}{cc} A_{11}^{a} & =M_{U|{\tilde{Y}}_{1}}\left(G_{1}B_{1}+I\right),\;A_{12}^{a}=M_{U|{\tilde{Y}}_{1}}G_{1}B_{2}. \end{array}$$

With the above choice of $\left(A_{11},A_{12}\right)$, we have

$$h\left(U|X_{1},Y_{1}\right)=h\left(X_{01}^{\prime }-M_{U|{\tilde{Y}}_{1}}\left(G_{1}\left(X_{01}^{\prime }+X_{02}^{\prime }\right)+Z_{1}\right)\right).$$

Finally, we demand that $M_{U|{\tilde{Y}}_{1}}$ be the MMSE of $X_{01}^{\prime }$ given $G_{1}\left(X_{01}^{\prime }+X_{02}^{\prime }\right)+Z_{1}$, i.e.,

$$M_{U|{\tilde{Y}}_{1}}={\left(G_{1}\left(K_{01}^{\prime }+K_{02}^{\prime }\right)G_{1}^{T}+I\right)}^{-1}K_{01}^{\prime }G_{1}^{T}.$$

In such case

$$\begin{array}{cc} A_{11}^{a} & ={\left(G_{1}\left(K_{01}^{\prime }+K_{02}^{\prime }\right)G_{1}^{T}+I\right)}^{-1}K_{01}^{\prime }G_{1}^{T}\left(G_{1}B_{1}+I\right),\\ A_{12}^{a} & ={\left(G_{1}\left(K_{01}^{\prime }+K_{02}^{\prime }\right)G_{1}^{T}+I\right)}^{-1}K_{01}^{\prime }G_{1}^{T}G_{1}B_{2}. \end{array}$$

Hence

$$h\left(U|X_{1},Y_{1}\right)=h\left(X_{01}^{\prime }|G_{1}\left(X_{01}^{\prime }+X_{02}^{\prime }\right)+Z_{1}\right),$$

and thus

$$\begin{array}{cc} h\left(U|S,X_{1}\right)-h\left(U|X_{1},Y_{1}\right) & =h\left(X_{01}^{\prime }\right)-h\left(X_{01}^{\prime }|G_{1}\left(X_{01}^{\prime }+X_{02}^{\prime }\right)+Z_{1}\right)\\ & =I\left(X_{01}^{\prime };G_{1}\left(X_{01}^{\prime }+X_{02}^{\prime }\right)+Z_{1}\right)\\ & =\frac{1}{2}\log\left(\frac{|G_{1}\left(K_{01}^{\prime }+K_{02}^{\prime }\right)G_{1}^{T}+I|}{|G_{1}K_{02}^{\prime }G_{1}^{T}+I|}\right). \end{array}$$

Furthermore, if we choose $A_{11}^{b}=B_{1}+G_{1}^{-1}$ and $A_{12}^{b}=B_{2}$, then

$$\begin{array}{cc} I(X_{1};Y_{1}|U) & =I\left(X_{1};G_{1}\left(X_{01}^{\prime }+X_{02}^{\prime }+B_{1}S_{1}+B_{2}S_{2}\right)+X_{1}+S_{1}+Z_{1}|X_{01}^{\prime }+A_{11}^{b}S_{1}+A_{12}^{b}S_{2}\right)\\ & =I(X_{1};G_{1}X_{02}^{\prime }+X_{1}+Z_{1})\\ & =\frac{1}{2}\log\left(\frac{|G_{1}K_{02}G_{1}^{T}+K_{1}+I|}{|G_{1}K_{02}G_{1}^{T}+I|}\right). \end{array}$$

We next consider the bound on $R_{2}$. Consider the first argument in min of (28b)

(A12) $$\begin{array}{cc} I\left(V,X_{2};Y_{2}\right)-I\left(V;U,S\right) & =I\left(X_{2};Y_{2}\right)+I\left(V;Y_{2}|X_{2}\right)-I\left(V;U,S|X_{2}\right)\\ & =I\left(X_{2};Y_{2}\right)+h\left(V|U,S,X_{2}\right)-h\left(V|Y_{2},X_{2}\right). \end{array}$$

It is straightforward to show that

$$\begin{array}{c} I\left(X_{2};Y_{2}\right)=\frac{1}{2}\log\left(\frac{|G_{2}K_{0}G_{2}^{T}+K_{2}+G_{2}B_{2}K_{S_{2}}+K_{S_{2}}B_{2}^{T}G_{2}^{T}+K_{S_{2}}+I|}{|G_{2}K_{0}G_{2}^{T}+G_{2}B_{2}K_{S_{2}}+K_{S_{2}}B_{2}^{T}G_{2}^{T}+K_{S_{2}}+I|}\right), \end{array}$$

and $h\left(V|U,S,X_{2}\right)=h\left(X_{02}^{\prime }\right)$. As for the third term in (A12), denote ${\tilde{Y}}_{2}=Y_{2}-X_{2}$

$$\begin{array}{cc} h(V|Y_{2},X_{2}) & =h\left(V|{\tilde{Y}}_{2}\right)=h\left(V-M_{V|{\tilde{Y}}_{2}}{\tilde{Y}}_{2}\right)\\ & =h\left(X_{02}^{\prime }+A_{20}X_{01}^{\prime }+A_{21}S_{1}+A_{22}S_{2}-M_{V|{\tilde{Y}}_{2}}\left(G_{2}\left(X_{01}^{\prime }+X_{02}^{\prime }+B_{1}S_{1}+B_{2}S_{2}\right)+S_{2}+Z_{2}\right)\right). \end{array}$$

We require that terms $X_{01}^{\prime }$, $S_{1}$ and $S_{2}$ in the argument of the differential entropy be completely canceled, hence we choose

$$\begin{array}{c} A_{20}^{a}=M_{V|{\tilde{Y}}_{2}}G_{2},\;A_{21}^{a}=M_{V|{\tilde{Y}}_{2}}G_{2}B_{1},\;A_{12}^{a}=M_{V|{\tilde{Y}}_{2}}\left(G_{2}B_{2}+I\right). \end{array}$$

With the above choice of $\left(A_{20},A_{21},A_{22}\right)$, we have

$$h\left(V|Y_{2},X_{2}\right)=h\left(X_{02}^{\prime }-M_{V|{\tilde{Y}}_{2}}\left(G_{2}X_{02}^{\prime }.+Z_{2}\right)\right).$$

Following this we demand that $M_{V|{\tilde{Y}}_{2}}$ be the MMSE of $X_{02}^{\prime }$ given $G_{2}X_{02}^{\prime }+Z_{2}$, i.e.,

$$M_{V|{\tilde{Y}}_{2}}={\left(G_{2}K_{02}G_{2}^{T}+I\right)}^{-1}K_{02}G_{2}^{T}.$$

In such case

$$\begin{array}{cc} A_{20}^{a} & ={\left(G_{2}K_{02}G_{2}^{T}+I\right)}^{-1}K_{02}G_{2}^{T}G_{2},\\ A_{21}^{a} & ={\left(G_{2}K_{02}G_{2}^{T}+I\right)}^{-1}K_{02}G_{2}^{T}G_{2}B_{1},\\ A_{22}^{a} & ={\left(G_{2}K_{02}G_{2}^{T}+I\right)}^{-1}K_{02}G_{2}^{T}\left(G_{2}B_{2}+I\right). \end{array}$$

Hence

$$h\left(V|Y_{2},X_{2}\right)=h\left(X_{02}^{\prime }|G_{2}X_{02}^{\prime }+Z_{2}\right),$$

and thus

$$\begin{array}{cc} h\left(V|U,S,X_{2}\right)-h\left(V|Y_{2},X_{2}\right) & =h\left(X_{02}^{\prime }\right)-h\left(X_{02}^{\prime }|G_{2}X_{02}^{\prime }+Z_{2}\right)\\ & =I(X_{02}^{\prime };G_{2}X_{02}^{\prime }+Z_{2})\\ & =\frac{1}{2}\log\left(|G_{2}K_{02}G_{2}^{T}+I|\right). \end{array}$$

Furthermore, if we choose $A_{20}^{b}=I$, $A_{21}^{b}=B_{1}$ and $A_{22}^{b}=B_{2}+G_{2}^{-1}$, then

$$\begin{array}{cc} I(X_{2};Y_{2}|V) & =I\left(X_{2};X_{2}+Z_{2}\right)=\frac{1}{2}\log\left(|K_{2}+I|\right). \end{array}$$
